# Construction of pseudorabies virus variant attenuated vaccine: codon deoptimization of *US3* and *UL56* genes based on PRV gE/TK deletion strain

**DOI:** 10.3389/fmicb.2023.1248573

**Published:** 2023-10-10

**Authors:** Mengwei Xu, Laixu Zhu, Aimin Ge, Yamei Liu, Saisai Chen, Ziwen Wei, Yating Zheng, Ling Tong, Zhisheng Wang, Rongmei Fei, Jichun Wang, Chuanjian Zhang

**Affiliations:** ^1^National Research Center of Engineering and Technology for Veterinary Biologicals, Jiangsu Key Laboratory for Food Quality and Safety-State Key Laboratory Cultivation Base of the Ministry of Science and Technology, Institute of Veterinary Immunology and Engineering, Jiangsu Academy of Agricultural Sciences, Nanjing, China; ^2^GuoTai (Taizhou) Center of Technology Innovation for Veterinary Biologicals, Taizhou, China; ^3^Jiangsu Co-Innovation Center for Prevention and Control of Important Animal Infectious Diseases and Zoonoses, Yangzhou, China; ^4^College of Veterinary Medicine, Nanjing Agricultural University, Nanjing, China; ^5^Shandong Vocational Animal Science and Veterinary College, Weifang, China

**Keywords:** pseudorabies virus, attenuation, immunogenicity, codon deoptimization, US3-S, UL56

## Abstract

Since 2011, pseudorabies based on the pseudorabies virus (PRV) variant has emerged as a serious health issue in pig farms in China. The PRV gE/TK or gE/gI/TK deletion strains protect against emerging PRV variants. However, these variants may cause lethal infections in newborn piglets without PRV antibodies. Previous studies have shown that codon deoptimization of a virulence gene causes virus attenuation. Accordingly, we deoptimized US3-S (US3 gene encoding a short isoform that represents approximately 95% of the total US3 transcription) and *UL56* genes (first 10 or all codons) of PRV gE/TK deletion strain (PRV^ΔTK&gE−AH02^) to generate six recombinant PRVs through bacterial artificial chromosome technology. In swine testicular cells, recombinant PRVs with all codon deoptimization of *US3-S* or *UL56* genes were grown to lower titers than the parental virus. Notably, US3-S or UL56 with all codon deoptimization reduced mRNA and protein expressions. Subsequently, the safety and immunogenicity of recombinant PRVs with codon deoptimization of US3-S or UL56 are evaluated as vaccine candidates in mice and piglets. The mice inoculated with recombinant PRVs with codon deoptimization of US3-S or UL56 showed exceptional survival ability without severe clinical signs. All codons deoptimized (US3-S and UL56) significantly decreased virus load and attenuated pathological changes in the brains of the mice. Moreover, the protection efficiency offered by recombinant PRVs with codon deoptimization of US3-S or UL56 showed similar effects to PRV^ΔTK&gE−AH02^. Remarkably, the 1-day-old PRV antibody-negative piglets inoculated with PRV^ΔTK&gE^-US3-S^T−CD^ (a recombinant PRV with all codon deoptimization of US3-S) presented no abnormal clinical symptoms, including fever. The piglets inoculated with PRV^ΔTK&gE^-US3-S^T−CD^ showed a high serum neutralization index against the PRV variant. In conclusion, these results suggest using codon deoptimization to generate innovative live attenuated PRV vaccine candidates.

## 1. Introduction

Swine pseudorabies, an acute disease caused by the pseudorabies virus (PRV), has emerged as the leading cause of fatal encephalitis in newborn piglets, respiratory illness and growth stagnation in the growing pigs, as well as reproductive failure in the sows (Pomeranz et al., 2005). After extensive research, it was increasingly recognized that the utilization of live attenuated PRV Bartha-K61 vaccine combined with gE-ELISA serologic differential diagnosis showed exceptional control in spreading PRV in China from the 1990s to 2010 (Freuling et al., 2017). Nevertheless, pseudorabies outbreaks since 2011 caused by PRV variants have occurred in various Bartha-K61-vaccinated swine herds in China (An et al., 2013; Yu et al., 2014). Previous studies indicated that the Bartha-K61 vaccine could not fully protect against PRV variants, especially in preventing virus shedding (Zhou et al., 2017; Zhang et al., 2019a). To overcome this issue, PRV gE/TK and TK/gE/gI deletion strains based on current PRV variants have been developed, presenting good immunogenicity in pigs against PRV variants (Zhang et al., 2015). Nonetheless, the safety of these PRV deletion vaccines for neonatal piglets is particularly perturbing, hampering their utilization (Wang et al., 2018). In our previous study, we generated a PRV gE/TK deletion strain (PRV^ΔTK&gE−AH02^) based on a virulent PRV AH02LA strain. This approach provided 100% clinical protection against the AH02LA strain in weaned pigs. However, the experimental results showed a lethal infection in newborn piglets without PRV antibodies (Wang et al., 2018). Therefore, further attenuation of PRV^ΔTK&gE−AH02^ while maintaining immunogenicity is necessary for developing a safe and effective live PRV vaccine.

Most amino acids (except methionine and tryptophan) in organisms are typically coded by synonymous codons (Knight et al., 2001). Notably, these synonymous codons display the same coding potential. However, most species show a codon usage bias in their protein-encoding genes (Kanaya et al., 2001; Knight et al., 2001). Considering the codon usage bias, strategies such as codon optimization or codon deoptimization have been applied to increase or decrease gene expression in different organisms, respectively. To this end, the codon deoptimization strategy is often achieved by replacing original codons with less-preferred usage codons, which do not affect the amino acid sequence of the protein or its function. However, it decreases protein production of recoded genes during transcription and translation at multiple levels (Goncalves-Carneiro and Bieniasz, 2021). Previous studies indicated that codon deoptimization enabled the highly efficient attenuation of RNA viruses (influenza A virus, foot-and-mouth disease virus, and lassa virus) and DNA viruses (vaccinia virus) by reducing the gene expression of a particular viral gene (Nogales et al., 2014; Diaz-San Segundo et al., 2016; Cai et al., 2020; Lorenzo et al., 2022).

The *US3* gene, a virulence gene of PRV, encodes two isoforms of pUS3 (Olsen et al., 2006). The larger US3-L transcript represents approximately 5% of the total US3 transcription, and the smaller US3-S transcript represents about 95% of the total US3 transcription (Sehl et al., 2020). The *UL56* gene, an encoding envelope protein, is an important virulence factor of PRV, enhancing virus spread and pathogenesis (Daniel et al., 2016). In several instances, we demonstrated that the inactivated US3-S or UL56 of PRV (a gene point mutant in the start codon to stop the expression of US3-S or UL56) displayed significantly attenuated virulence in mice (Lv et al., 2020a,b). However, the US3 deletion showed a decrease in PRV immunogenicity in pigs. In a case study, Xu and colleagues demonstrated that the *US3* gene deletion resulted in further attenuation of the PRV TK/gE deletion mutant (Xu et al., 2022). However, the immunogenicity of the PRV PK/TK/gE deletion variant strain was not stable (data not published). Similarly, a previous study showed that the protective efficiency of the PRV PK/gE deletion mutant was lower than that of the PRV gE deletion mutant in pigs (Kimman et al., 1994).

Considering these aspects, this study is aimed to demonstrate the construction of six recombinant PRVs harboring the deoptimized *US3-S* and *UL56* genes (first 10 or all codon deoptimization) based on PRV ΔgE/TK strain using a bacterial artificial chromosome (BAC) technology. Moreover, the experiments are carried out in mice and piglets to determine the pathogenicity and immunogenicity characteristics of recombinant PRVs.

## 2. Materials and methods

### 2.1. Condon deoptimization of *US3* and *UL56* genes

The first 10 codons, or all codons of *US3-S* and *UL56* genes, were recoded by rearranging the synonymous codons to minimize the cumulative codon scores based on the pig codon pair bias table. The recoded sequences were synthesized by Beijing Tsingke Biotech Co., Ltd., (Beijing, China) and cloned into the pMD19-T simple vector (Takara Bio Inc., Tokyo, Japan), named US3-S^F10−CD^-T, US3-S^T−CD^-T, UL56 ^F10−CD^-T, and UL56^T−CD^-T.

### 2.2. Viruses, cells, and plasmids

In this study, a PRV variant strain, AH02LA, was isolated from the brain of a dead newborn piglet in Anhui Province by our laboratory (CGMCC No. 10891). In addition, PRV^ΔTK&gE−AH02^ with TK/gE deletion based on the AH02LA strain was previously constructed in our lab (Wang et al., 2018). Furthermore, it should be noted that all viruses were transfected and propagated in swine testicular (ST) cells. The ST cells were cultured in Dulbecco's Modified Eagle Medium (DMEM, Gibco, USA) supplemented with 2 or 10% newborn calf serum (Gibco) and 1% penicillin and streptomycin (Sigma-Aldrich, St. Louis, USA) at 37°C under 5% CO_2_ atmosphere. On the one hand, the wild-type and recoded US3-S and UL56 were cloned into pmKate2-N plasmid at *Eco*R I restriction sites to construct the pUS3-S-mKate2-N and pUL56-mKate2-N plasmids, respectively. On the other hand, the TK/gE/gI-deleted PRV BAC (pPRV^Δ*TK&gE&gI*^) in which gI and gE genes were replaced with mini-F was constructed in our lab as reported previously (Wang et al., 2018). Furthermore, a kanamycin resistance gene was respectively inserted in US3-S^F10−CD^-T, US3-S^T−CD^-T, UL56 ^F10−CD^-T, and UL56^T−CD^-T, at the *Xma* I restriction site of US3-S^F10−CD^-T, *Xba* I restriction site of US3-S^T−CD^-T, *Nae* I restriction site of UL56^F10−CD^-T, and *Eco*R V restriction site of UL56^T−CD^-T to construct *En Passant* recombination.

### 2.3. Bacterial gene manipulation, polymerase chain reaction, and sequencing

As mentioned in the previous section, US3-S^F10−CD^-KAN, US3-S^T−CD^-KAN, UL56 ^F10−CD^-KAN, and UL56^T−CD^-KAN with 40-bp homologous sequences of PRV in both terminals were initially amplified with primers of US3-S^F10−CD^ En pa F/R, US3-S^T−CD^ En pa F/R, UL56 ^F10−CD^ En pa F/R, and UL56^T−CD^ En pa F/R (Supplementary Table S1) from US3-S^F10−CD^-T-KAN, US3-S^T−CD^-T-KAN, UL56 ^F10−CD^-T-KAN, and UL56^T−CD^-T-KAN. After digestion with *Dpn* I, four PCR products were respectively electroporated into GS1783 with pPRV^ΔTK&gE&gI^ to achieve the primary recombination, and a subsequent secondary red recombination resulted in the removal of the kanamycin resistance gene (Tischer et al., 2010). Four recombinant target clones (pPRV^ΔTK&gE&gI^-US3-S^F10−CD^-mini-F, pPRV^ΔTK&gE&gI^-US3-S^T−CD^-mini-F, pPRV^ΔTK&gE&gI^-UL56^F10−CD^-mini-F, and pPRV^ΔTK&gE&gI^-UL56^T−CD^-mini-F) were successfully generated. Furthermore, pPRV^ΔTK&gE&gI^-US3-S&UL56^F10−CD^-mini-F and pPRV^ΔTK&gE&gI^-US3-S&UL56^T−CD^-mini-F were constructed based on pPRV^ΔTK&gE&gI^-US3-S^F10−CD^-mini-F and pPRV^ΔTK&gE&gI^-US3-S^T−CD^-mini-F by *En Passant* recombination. Eventually, these generated six clones were confirmed by restriction fragment length polymorphism (RFLP) with *Bam*H I. The recoded US3-S and UL56 were identified through PCR and sequencing.

### 2.4. Generation of recombinant viruses

Briefly, the primers of H1-H2-gI-ΔgE F/R (Supplementary Table S1) were designed to amplify H1-H2-gI-ΔgE (the whole gI gene and part of gE gene with homologous arms at both ends) from PRV^ΔTK&gE−AH02^. Then, to recover infectious viruses, 1-μg pPRV^ΔTK&gE&gI^-US3-S^F10−CD^-mini-F, pPRV^ΔTK&gE&gI^-UL56^F10−CD^-mini-F, pPRV^ΔTK&gE&gI^-US3-S&UL56^F10−CD^-mini-F, pPRV^ΔTK&gE&gI^-US3-S^T−CD^-mini-F, pPRV^ΔTK&gE&gI^-UL56^T−CD^-mini-F, or pPRV^ΔTK&gE&gI^-US3-S&UL56^T−CD^-mini-F and 1-μg H1-H2-gI-ΔgE were co-transfected into ST cells with Lipofectamine^®^ 3000 reagent (Invitrogen, Waltham, USA) according to the manufacturer's instructions. After 24–48 h of transfection, fluorescent plaques (recombinant PRVs from BAC) and non-fluorescent plaques (gI-ΔgE-recovered PRVs in which the mini-F sequences were replaced with the whole gI gene and part of gE gene) were observed. After three rounds of plaque purification, the resultant non-fluorescent plaques were purified and named PRV^ΔTK&gE^-US3-S^F10−CD^ (PRV TK/gE double gene deletion strain with first 10 codon deoptimization of US3-S), PRV^ΔTK&gE^-UL56^F10−CD^ (PRV TK/gE double gene deletion strain with first 10 codon deoptimization of UL56), PRV^ΔTK&gE^-US3-S&UL56^F10−CD^ (PRV TK/gE double gene deletion strain with first 10 codon deoptimization of US3-S and UL56), PRV^ΔTK&gE^-US3-S^T−CD^ (PRV TK/gE double gene deletion strain with all codon deoptimization of US3-S), PRV^ΔTK&gE^-UL56^T−CD^ (PRV TK/gE double gene deletion strain with all codon deoptimization of UL56), and PRV^ΔTK&gE^-US3-S&UL56^T−CD^ (PRV TK/gE double gene deletion strain with all codon deoptimization of US3-S and UL56). The recombinant viruses were cultured by passaging 20 times on ST cells. Finally, the recoded US3-S and UL56 were confirmed with PCR and sequencing experimentation.

### 2.5. Multi-step growth kinetics of recombinant viruses

To explore the multi-step growth kinetics of recombinant viruses, ST cells were infected with PRV^ΔTK&gE−AH02^ and the before-mentioned six recombinant viruses at a multiplicity of infection (MOI) of 0.01. At 6, 12, 24, 36, 48, 60, and 72 h post-infection, the culture cells were harvested and titrated on monolayers of ST cells. It should be noted that the experiments were performed in triplicate and analyzed using a one-way ANOVA by SPSS 16.0 (SPSS Inc., Chicago, USA).

### 2.6. RNA and protein expressions of codon-deoptimized *US3-S* and *UL56* genes

Briefly, 1.5 μg of expression plasmids, i.e., pUS3-S-mKate2-N, pUS3-S^F10−CD^-mKate2-N, pUS3-S^T−CD^-mKate2, pUL56-mKate2, pUL56^F10−CD^-mKate2, or pUL56^T−CD^-mKate2, were initially transfected into ST cells. At 24 h post-infection, the total RNA of the transfected cells was isolated using the TRIzol reagent (Sigma-Aldrich) (Chomczynski and Sacchi, 1987). Furthermore, 1 μg of total RNA was reverse transcribed with a PrimeScript^®^ RT Reagent Kit with gDNA Eraser (Takara Co. Ltd.). Then, the gene-specific primers were used to quantify the transcripts of US3-S and UL56 (Supplementary Table S1). The real-time quantitative PCR (qRT-PCR) was carried out on the Roche Light Cycler^®^ 480 system (Roche Diagnostics, Burgess Hill, UK) using SYBR Premix Ex Taq dye (Takara Co. Ltd.) (Zhang et al., 2019b). Notably, each cDNA was analyzed in triplicate, and sample data were normalized to β-actin expression using the 2^−Δ*ΔCt*^ method.

Furthermore, transcription of the recoded US3-S and UL56 genes in the viral background was assessed as follows: Briefly, ST cells were seeded in a 6-well plate and then infected with PRV^ΔTK&gE−AH02^ or six recombinant viruses at an MOI of 1. At 12 and 24 h post-infection, early genes (US3-S, UL40, and UL52), as well as late genes (UL24, UL44, and UL56) mRNA, were quantified by qRT-PCR using the gene-specific primers (Supplementary Table S1). Then, RNA extraction, reverse transcription, and qRT-PCR were performed as described above.

Then, the protein production of recoded US3-S and UL56 was analyzed using the following procedure: ST cells were initially transfected with 1.5 μg of the pmKate2-N, pUS3-S-mKate2-N, pUS3-S^F10−CD^-mKate2-N, pUS3-S^T−CD^-mKate2-N, pUL56^F10−CD^-mKate2-N, pUL56^T−CD^-mKate2-N, or pUL56-mKate2-N plasmids. At 24 h post-transfection, cells were fixed with 4% paraformaldehyde for 30 min and dyed with 4′,6-diamidino-2-phenylindole (DAPI; Sigma-Aldrich) for 20 min at room temperature (Weng et al., 2023). An inverted fluorescence microscope was used to examine transfected cells, and the representative cells were photographed.

### 2.7. Pathogenicity and immunological experiments in mice

Imprinting control region, healthy female mice (age of 6 weeks, *n* = 286) were purchased from Shanghai Laboratory Animal Co. Ltd., (SLAC, Shanghai, China). Initially, the purchased animals were randomly divided into 22 groups (*n* = 13 in each group). Mice were subcutaneously inoculated with 10^6.5^ TCID_50_, 10^5.5^ TCID_50_, and 10^4.5^ TCID_50_ PRV^ΔTK&gE^-US3-S^F10−CD^, PRV^ΔTK&gE^-UL56^F10−CD^, PRV^ΔTK&gE^-US3-S&UL56^F10−CD^, PRV^ΔTK&gE^-US3-S^T−CD^, PRV^ΔTK&gE^-UL56^T−CD^, PRV^ΔTK&gE^-US3-S&UL56^T−CD^, or PRV^ΔTK&gE−AH02^, and inoculated with DMEM serving as a negative control. The clinical symptoms and mortality of mice were observed daily for 14 days. On day 5 post-inoculation, five mice from each group were sacrificed, and the brain and lung samples were collected. Furthermore, the viral loads in the brain and lung samples of the sacrificed mice were detected using qRT-PCR analysis of the PRV gB in the Roche Light Cycler^®^ 480 system (Roche Diagnostics, Burgess Hill, UK) as described previously (Zhang et al., 2019b). Then, the PRV copy numbers in the brain and lung samples were expressed as log10 copies per gram of tissue sample. To this end, the brain and lung tissues were fixed using 4% paraformaldehyde for 24 h. The fixed tissue samples were embedded in paraffin wax and cut into 3 μm sections. The tissue sections were stained with hematoxylin and eosin and examined by light microscopy. In addition, on day 21 post-inoculation, all surviving mice were confronted with 100 LD_50_ PRV AH02LA strain, and the clinical symptoms and mortality rate of mice were monitored daily for 14 days.

### 2.8. Pathogenicity and immunological experiments in piglets

To explore the pathogenicity and immunogenicity in pigs, 1-day-old piglets (*n* = 15) free of PRV, porcine reproductive and respiratory syndrome viruses, porcine parvovirus, and porcine circovirus 2 were considered. Initially, the randomly distributed piglets were intramuscularly administered with 1 ml PRV^ΔTK&gE^-US3-S^T−CD^ (10^5.00^ TCID_50_/ml) and PRV^ΔTK&gE−AH02^ (10^5.00^ TCID_50_/ml) and inoculated with DMEM serving as a negative control. Furthermore, the body temperature and clinical signs of all piglets were monitored daily for 14 days. To this end, serum samples were collected on days 7, 14, and 21 post-inoculation to monitor neutralizing antibody index. Then, 100 μl of serum sample (heat inactivated for 30 min at 56°C) was mixed with an equal volume of 10-fold-diluted AH02LA virus. The neutralization indexes were expressed as the TCID_50_ of serum in the test group divided by the TCID_50_ of serum in the control group.

### 2.9. Statistical analysis

Data were presented as mean ± standard error mean (SEM) and analyzed using a one-way analysis of variance (ANOVA) with a Tukey's *post-hoc* test (SPSS Inc., Chicago, IL, USA), considering the *p-*values of < 0.05 as statistically significant. ^*^ indicates *p* < 0.05, ^**^ represents *p* < 0.01, and ^***^ signifies *p* < 0.001.

## 3. Results

### 3.1. Codon deoptimization of US3-S and UL56

Initially, the first 10 codons, or all codons of the *US3-S* and *UL56* genes, were deoptimized based on the pig codon pair bias table without any alterations to the amino acid sequences. The nucleotide sequences of US3-S, US3-S^F10−CD^, US3-S^T−CD^, UL56, UL56^F10−CD^, and UL56^T−CD^ are shown in Supplementary material S1. Compared to the wild-type US3-S gene, US3-S^F10−CD^ (accession numbers: OR228539) contained 9 codon changes through 9 nucleotide substitutions, and US3-S^T−CD^ (accession numbers: OR228540) contained 277 codon changes through 279 nucleotide substitutions. Similarly, several substitutions were observed in the UL56 coding region. Compared to the wild-type UL56 gene, UL56^F10−CD^ (accession numbers: OR228541) contained 7 codon changes through 7 nucleotide substitutions, and UL56^T−CD^ (accession numbers: OR228542) contained 155 codon changes through 155 nucleotide substitutions. US3-S^F10−CD^, US3-S^T−CD^, UL56^F10−CD^, and UL56^T−CD^ were synthesized by Beijing Tsingke Biotech Co., Ltd., (Beijing, China).

### 3.2. Construction of recombinant viruses with US3-S and UL56 codon deoptimization

**Figure 1:**
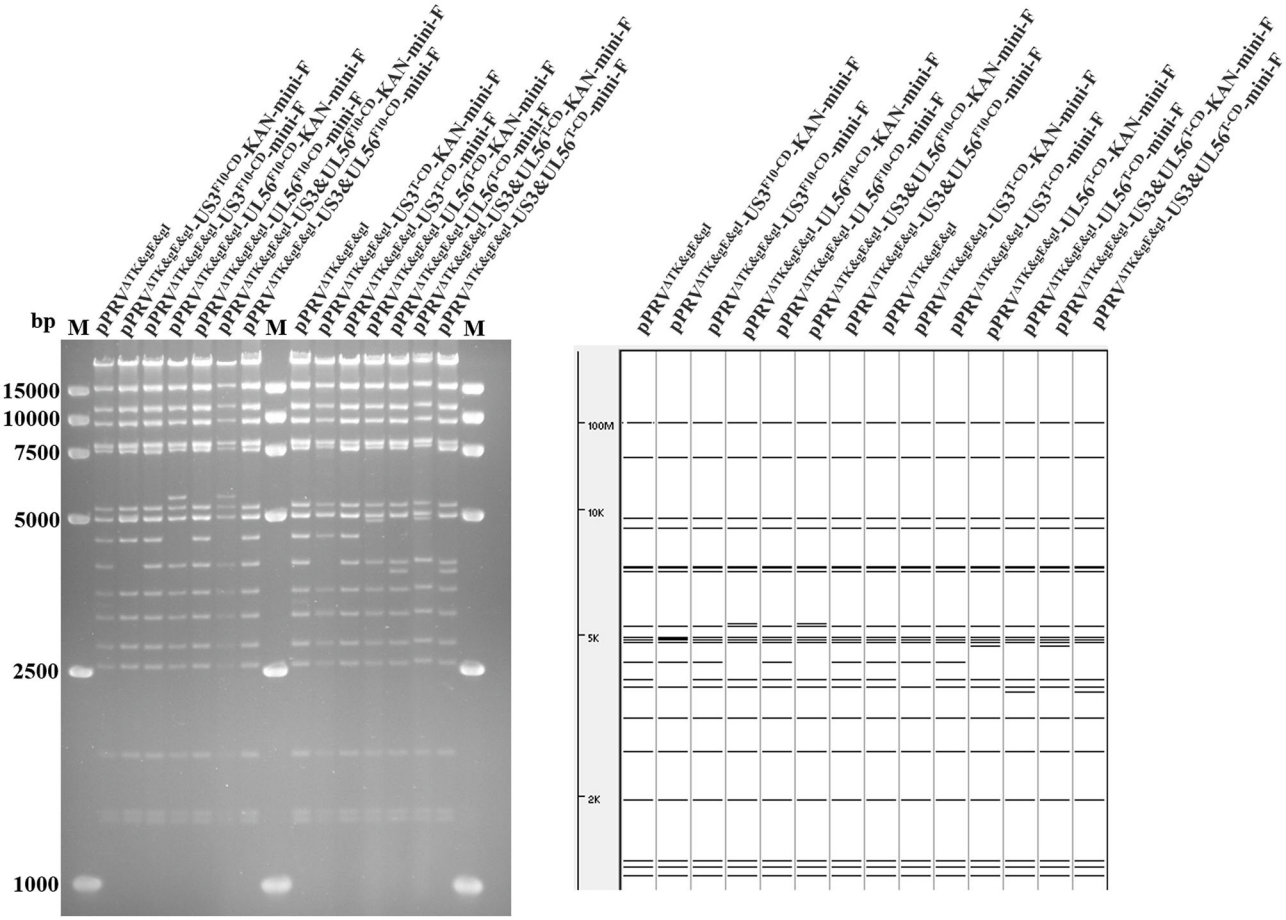
RFLP analysis shows the recombinant BACs with codon deoptimization of US3-S and UL56. Predictions of these digestions with *BamH* I using the PRV ZJ01 strain are performed (GenBank: KM061380.1).

**Figure 2:**
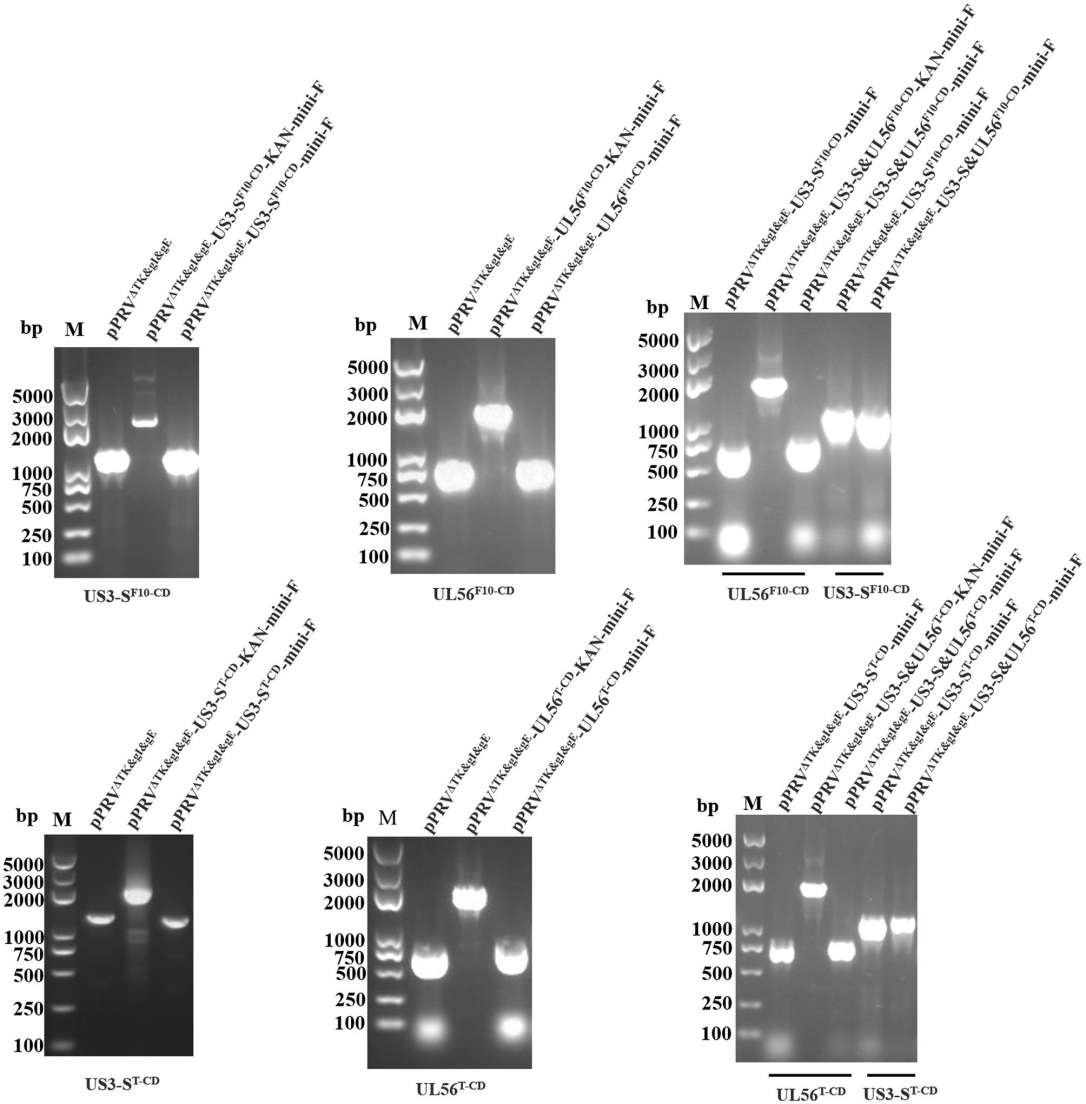
PCR identifies the recoded US3-S and UL56, the recoded US3-S with primers (US3-S check F/R), and the recoded UL56 with primers (UL56 check F/R) in the recombinant BACs.

**Figure 3:**
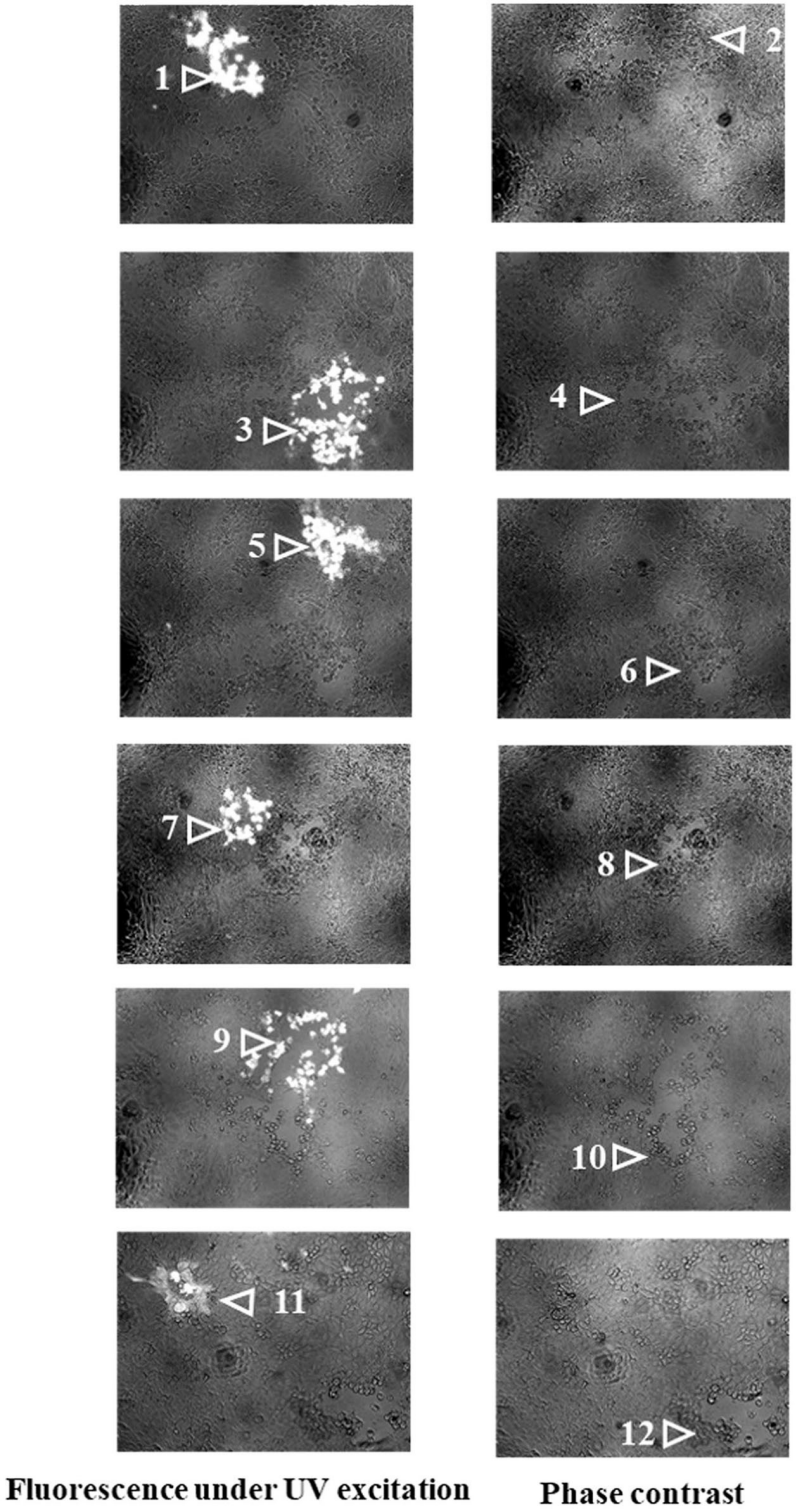
The images show recombinant PRVs with the recoded US3-S or UL56 genes under UV excitation and phase contrast. 1-μg pPRV^ΔTK&gE&gI^-US3-S^F10−CD^-mini-F, pPRV^ΔTK&gE&gI^-UL56^F10−CD^-mini-F, pPRV^ΔTK&gE&gI^-US3-S&UL56^F10−CD^-mini-F, pPRV^ΔTK&gE&gI^-US3-S^T−CD^-mini-F, pPRV^ΔTK&gE&gI^-UL56^T−CD^-mini-F, or pPRV^ΔTK&gE&gI^-US3-SandUL56^T−CD^-mini-F and 1-μg H1-H2-gI-ΔgE (the whole gI gene and part of gE gene with homologous arms at both ends) were co-transfected into ST cells. After 24 h of transfection, fluorescent plaques (recombinant PRVs from BAC) and non-fluorescent plaques (gI-ΔgE-recovered PRVs in which the mini-F sequences were replaced with the whole gI gene and part of the gE gene) were observed. Each panel represents a view of 200 × 200 μm in size. 1: PRV^ΔTK&gE&gI^-US3-S^F10−CD^-mini-F, 2: PRV^ΔTK&gE^-US3-S^F10−CD^, 3: PRV^ΔTK&gE&gI^-UL56^F10−CD^-mini-F, 4: PRV^ΔTK&gE^-UL56^F10−CD^, 5: PRV^ΔTK&gE&gI^-US3-S&UL56^F10−CD^-mini-F, 6: PRV^ΔTK&gE^-US3-SandUL56^F10−CD^, 7: PRV^ΔTK&gE&gI^-US3-S^T−CD^-mini-F, 8: PRV^ΔTK&gE^-US3-S^T−CD^, 9: PRV^ΔTK&gE&gI^-UL56^T−CD^-mini-F, 10: PRV^ΔTK&gE^-UL56^T−CD^, 11: PRV^ΔTK&gE&gI^-US3-SandUL56^T−CD^-mini-F, and 12: PRV^ΔTK&gE^-US3-SandUL56^T−CD^.

Based on pPRV^ΔTK&gE&gI^, *US3-S* and *UL56* genes were replaced with the recoded gene by *En Passant* recombination, generating six recombinant clones (pPRV^ΔTK&gE&gI^-US3-S^F10−CD^-mini-F, pPRV^ΔTK&gE&gI^-US3-S^T−CD^-mini-F, pPRV^ΔTK&gE&gI^-UL56^F10−CD^-mini-F, pPRV^ΔTK&gE&gI^-UL56^T−CD^-mini-F, pPRV^ΔTK&gE&gI^-US3-S&UL56^F10−CD^-mini-F, and pPRV^ΔTK&gE&gI^-US3-S&UL56^T−CD^-mini-F). Furthermore, it was observed that the RFLP analysis of six recombinant clones was slightly different from the predicted patterns after digestion with *Bam*H I (Figure 1). To validate these observations, the recoded US3-S and UL56 were confirmed by PCR and sequencing (Figure 2). To substantially generate recombinant PRVs with the recoded *US3-S* and *UL56* genes, the DNA of the six BACs and H1-H2-gI-ΔgE were co-transfected into ST cells. Furthermore, the resultant non-fluorescent plaques were observed under UV light at a wavelength of 488 nm at 24 h post-transfection (Figure 3). It should be noted that a homogeneous population of purified viruses was isolated by picking and plating for three rounds.

PCR and its sequencing analyses validated the correct sequences of recoded US3-S, recoded UL56, and H1-H2-gI-ΔgE (data not shown). The recombinant viruses were passaged 20 times on ST cells to investigate the genetic stability of gI-ΔgE (the whole gI gene and part of gE gene) and the recoded US3-S and UL56. Furthermore, the viral DNAs were extracted, and gI-ΔgE and the recoded US3-S and UL56 were detected by PCR and its sequencing analyses. It was observed from the PCR analysis that there was no change in gI-ΔgE and the recoded US3-S and UL56 (Supplementary Figure S1).

### 3.3. Multi-step growth kinetics of recombinant viruses with US3-S and UL56 codon deoptimization

**Figure 4:**
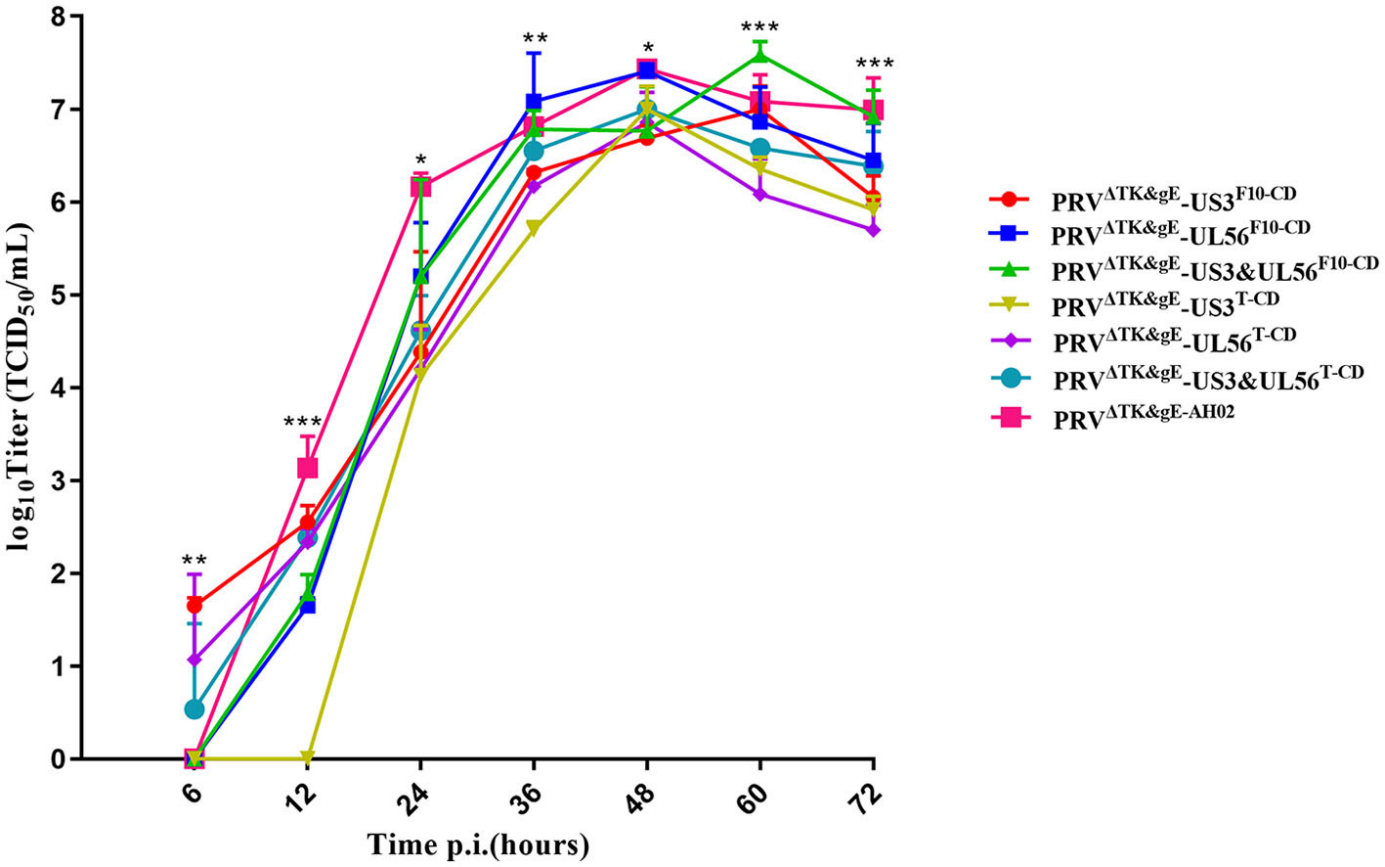
The multi-step growth curves indicate recombinant PRVs with the recoded US3-S or UL56 gene. ST cells are infected with PRV^ΔTK&gE−AH02^ and its six mutants at an MOI of 0.01. At 6, 12, 24, 36, 48, 60, and 72 h post-infection, the culture cells are harvested and titrated in ST cells. Asterisks indicate statistical significance among seven viruses (* indicates *P* < 0.05, ** represents *P* < 0.01, and *** signifies *P* < 0.001). Data are presented as mean ± SEM and analyzed using a one-way ANOVA with a Tukey's *post-hoc* test (SPSS Inc., Chicago, IL, USA).

The growth kinetics of the PRV^ΔTK&gE^-US3-S^F10−CD^, PRV^ΔTK&gE^-UL56^F10−CD^, PRV^ΔTK&gE^-US3-S&UL56^F10−CD^, PRV^ΔTK&gE^-US3-S^T−CD^, PRV^ΔTK&gE^-UL56^T−CD^, PRV^ΔTK&gE^-US3-S&UL56^T−CD^, and PRV^ΔTK&gE−AH02^ on ST cells are shown in Figure 4 and Supplementary Table S2. It was observed from the experimental results that PRV^ΔTK&gE^-US3-S^T−CD^, PRV^ΔTK&gE^-UL56^T−CD^, and PRV^ΔTK&gE^-US3-S&UL56^T−CD^ grew to lower titers than PRV^ΔTK&gE−AH02^, indicating that all codon deoptimization of US3-S or UL56 significantly affected the replication of the parental virus. Moreover, at 36 and 60 h post-infection, the titers of recombinant PRVs with the first 10 codon deoptimization of US3-S (10^6.32 ± 0.04^ TCID_50_/ml at 36 h post-infection; 10^7.00 ± 0.14^ TCID_50_/ml at 60 h post-infection) or UL56 (10^7.08 ± 0.30^ TCID_50_/ml at 36 h post-infection; 10^6.86 ± 0.22^ TCID_50_/ml at 60 h post-infection) were higher than recombinant PRVs with all codon deoptimization of US3-S (10^5.70 ± 0.05^ TCID_50_/ml at 36 h post-infection; 10^6.35 ± 0.08^ TCID_50_/ml at 60 h post-infection) or UL56 (10^6.17 ± 0.10^ TCID_50_/ml at 36 h post-infection; 10^6.08 ± 0.22^ TCID_50_/ml at 60 h post-infection).

### 3.4. Effect of codon deoptimization on mRNA and protein expression levels of recoded genes

**Figure 5:**
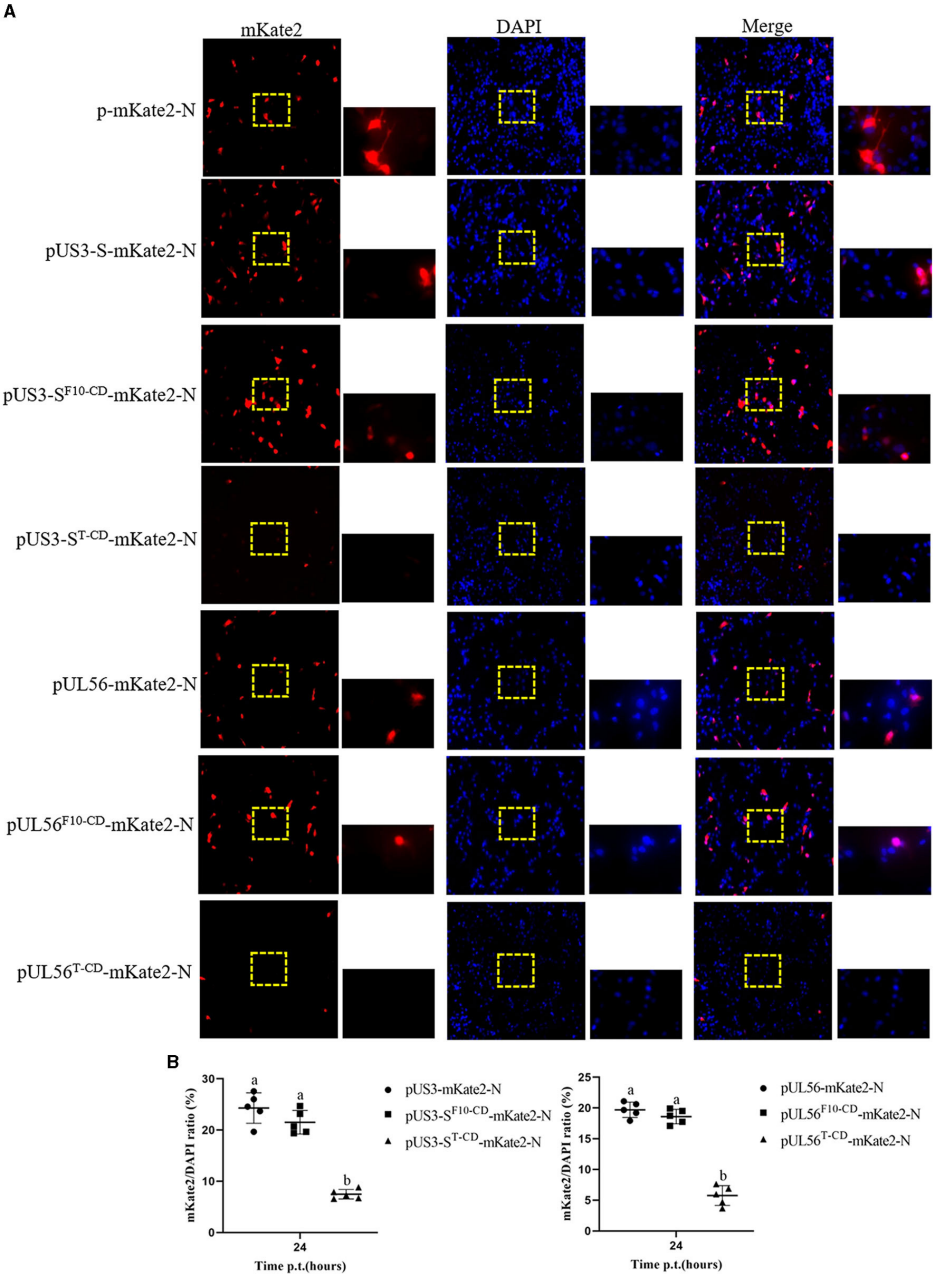
Protein expression from the recoded US3-S or UL56. **(A)** The images present ST transfected with recoded US3-S-mKate2 fusion or UL56-mKate2 fusion genes (200× and 800× magnification). **(B)** Quantitative analysis of the mKate2/DAPI ratio. Letters (a, b) above the bars indicate statistical significance (*P* < 0.05) of mKate2/DAPI ratio among the three plasmids.

**Figure 6:**
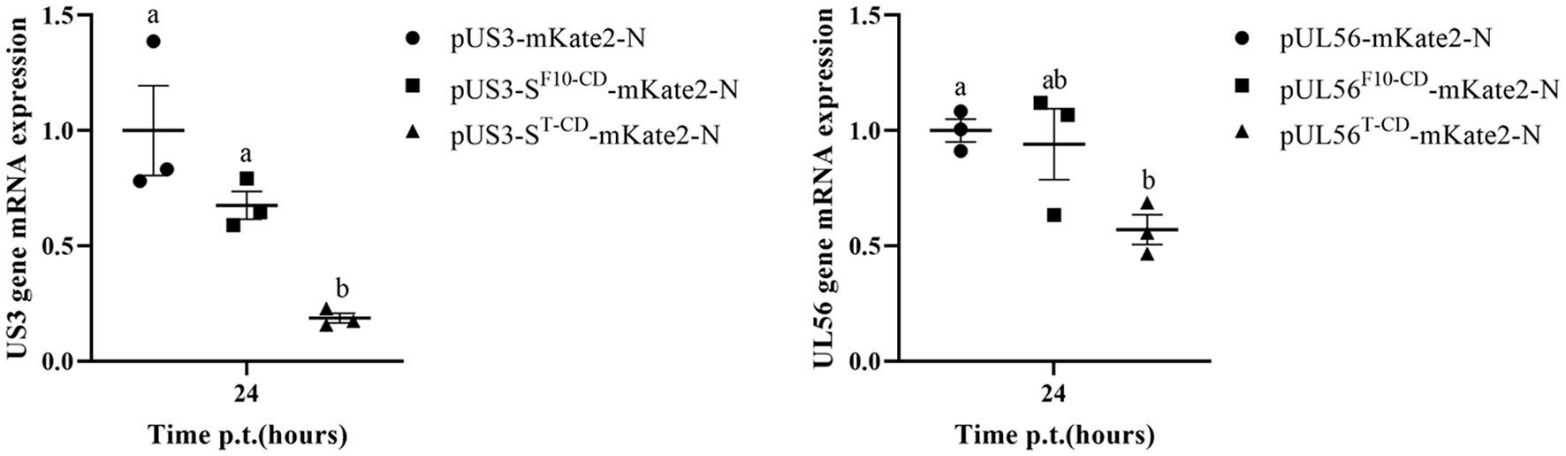
The quantification data show the RNA expression from the recoded *US3* gene or *UL56* gene. ST cells are transfected with the recoded US3-mKate2 fusion or UL56-mKate2 fusion genes. RNA expression from the recoded genes is quantified by qRT-PCR at 24 h post-transfection. Letters (a, b) above the bars indicate statistical significance (*P* < 0.05) of RNA expression among the three plasmids.

Furthermore, the effect of codon deoptimization on US3-S and UL56 protein production was evaluated. To explore this aspect, we constructed the expression plasmids pUS3-S-mKate2-N and pUL56-mKate2-N, in which US3-S and UL56 expressions were determined by the immediate-early promoter of human cytomegalovirus. In addition, the *US3-S* and *UL56* genes were C-terminally tagged with mKate2. Then, the plasmids containing US3-S, US3-S^F10−CD^, US3-S^T−CD^, UL56, UL56^F10−CD^, or UL56^T−CD^ were transfected into ST cells (Figure 5A). The mKate2/DAPI ratios of pUS3^T − CD^-mKate2-N and pUL56^T−CD^-mKate2-N were lower than their parental construct (Figure 5B). qRT-PCR analysis showed that the mRNA level of recoded genes in pUS3-S^T−CD^-mKate2-N and pUL56^T−CD^-mKate2-N was lower than their parental construct (Figure 6).

**Figure 7:**
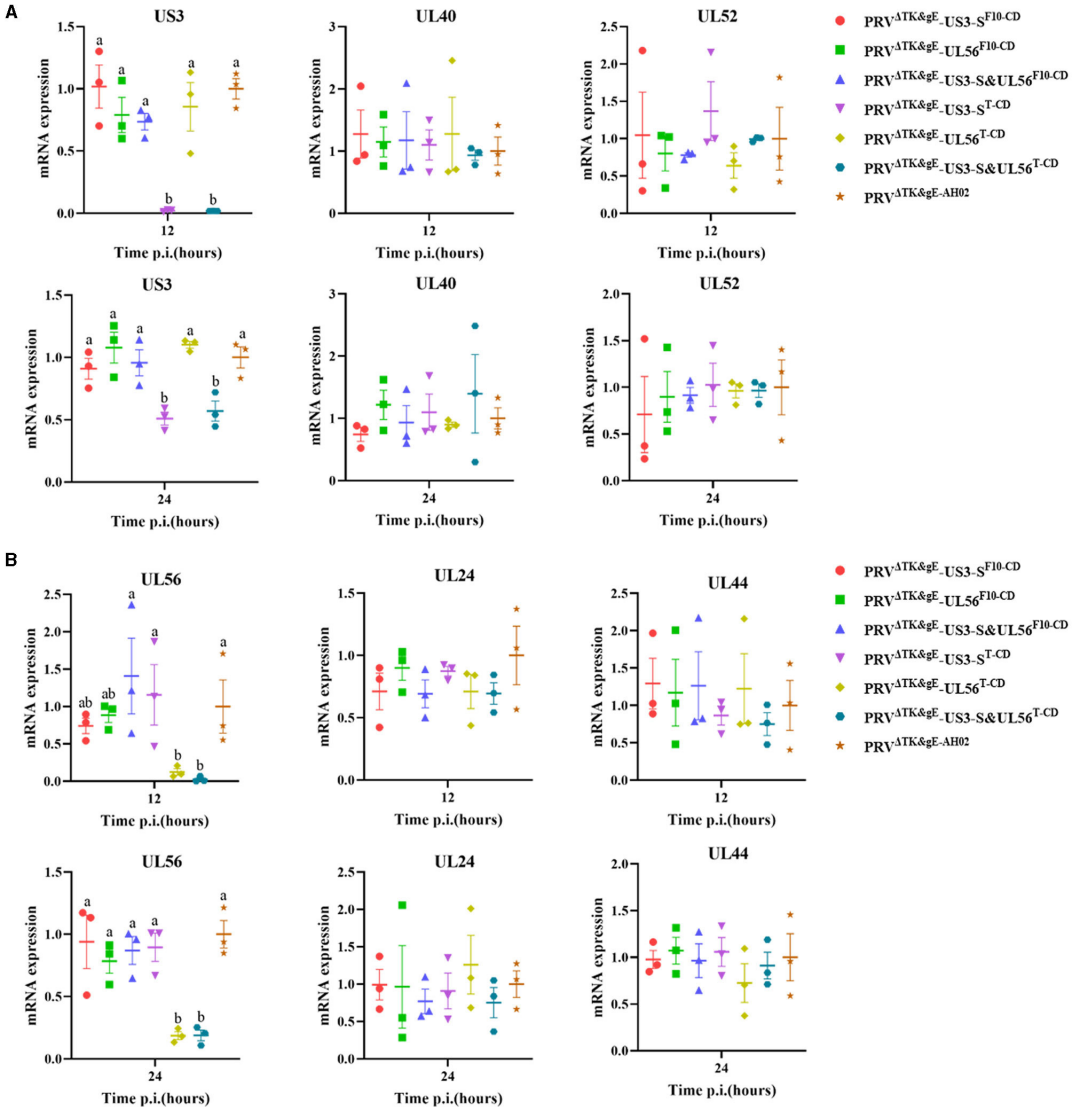
The data show the effect of recoding on early genes **(A)** or late genes **(B)** expression from the virus background. ST cells are infected with the parental or mutant virus that carries differently recoded *US3-S* or *UL56* genes. PCR quantifies mRNA expression of early genes (*US3, UL40*, and *UL52*) and late genes (*UL24, UL44*, and *UL56*) at 12 and 24 h post-infection. Letters (a, b) above the bars indicate statistical significance (*P* < 0.05) of RNA expression among seven viruses.

In addition, the RNA expression levels of US3-S, UL40, UL52, UL24, UL44, and UL56 during virus replication were determined (Figure 7). US3-S, UL40, and UL52 possessed the same kinetic gene expression class, representing early genes. UL24, UL44, and UL56 fitted to late genes. The quantification of RNA expression showed that all codon deoptimization negatively affected the mRNA expression of the recoded genes during virus replication.

### 3.5. Safety and immunogenicity of recombinant viruses with codon deoptimization of US3 or UL56 in mice and piglets

**Figure 8:**
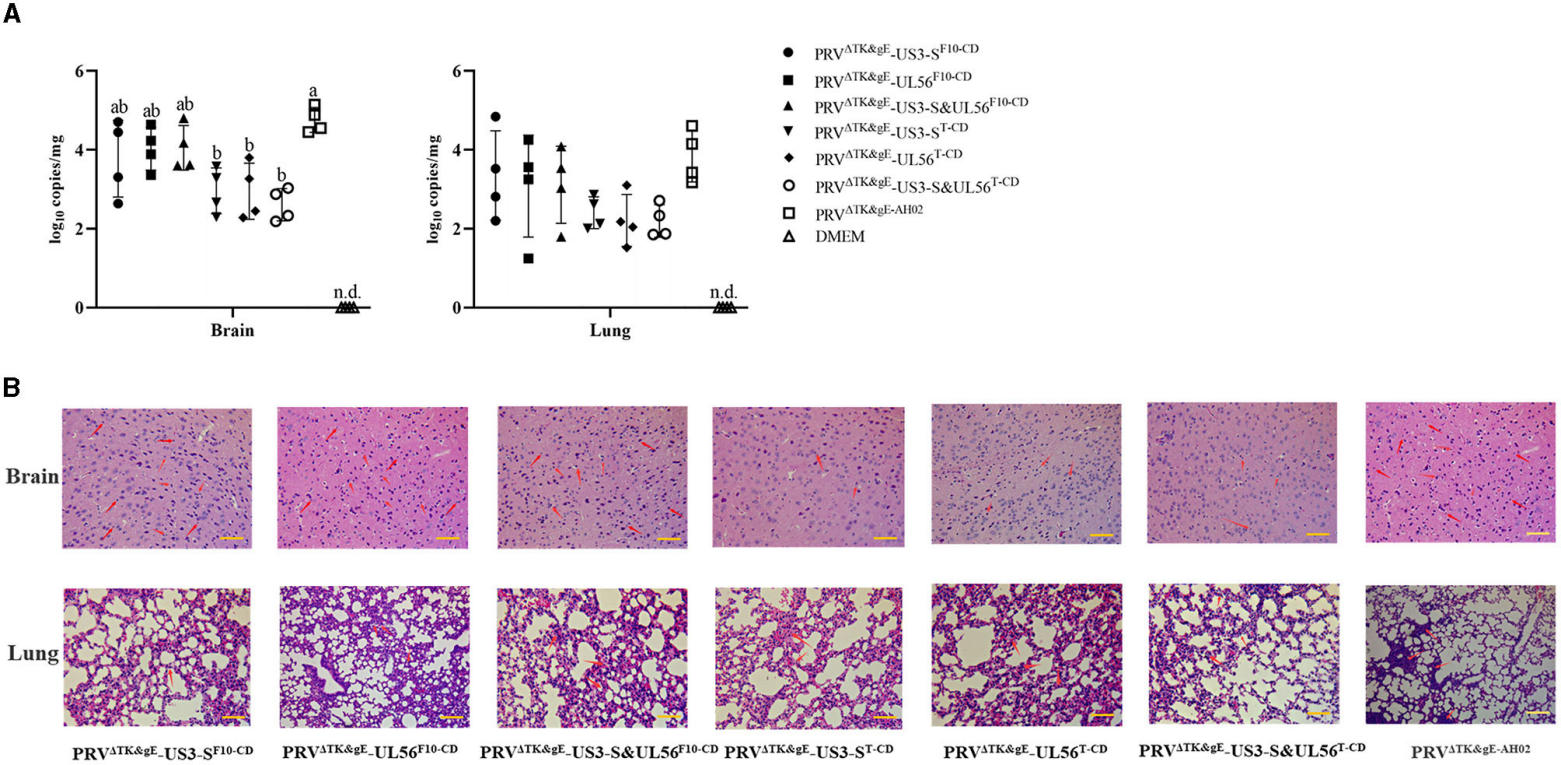
The graphs display the viral DNA loads quantification **(A)** and histological analysis **(B)** in the brain and lung of mice inoculated with 10^6.5^ TCID_50_ PRV^ΔTK&gE−AH02^ or recombinant PRVs with the recoded US3-S or UL56. Letters (a, b) above the bars indicate statistical significance (*P* < 0.05) of DNA loads among the eight groups. Arrows show inflammatory cell infiltration in the brain and lung (hematoxylin and eosin staining, 200× magnification, bar = 10 μm).

Eventually, the biological safety and significant immunogenicity of the designed recombinant virus were determined using mice and piglets. Mice inoculated with 10^6.5^ TCID_50_, 10^5.5^ TCID_50_, and 10^4.5^ TCID_50_ PRV^ΔTK&gE^-US3-S^F10−CD^, PRV^ΔTK&gE^-UL56^F10−CD^, PRV^ΔTK&gE^-US3-S&UL56^F10−CD^, PRV^ΔTK&gE^-US3-S^T−CD^, PRV^ΔTK&gE^-UL56^T−CD^, PRV^ΔTK&gE^-US3-S&UL56^T−CD^, PRV^ΔTK&gE−AH02^, and DMEM were survived without clinical symptoms, indicating no signs of toxicity and presenting excellent biosafety. On day 5 post-inoculation, mice inoculated with 10^6.5^ TCID_50_ recombinant PRVs with all codon deoptimization of US3-S and UL56 showed lower virus load in the brain compared with those inoculated with 10^6.5^ TCID_50_ PRV^ΔTK&gE−AH02^ (Figure 8A). In addition, no virus was detected in the brains and lungs of the mice inoculated with 10^5.5^ TCID_50_ and 10^4.5^ TCID_50_ PRV^ΔTK&gE−AH02^ or recombinant PRVs with codon deoptimization of US3-S or UL56. Furthermore, the histopathological examinations displayed that the mice inoculated with 10^6.5^ TCID_50_ PRV^ΔTK&gE−AH02^ showed obvious inflammatory cell infiltration in the brain. These abnormal cell infiltration consequences were more severe than those inoculated with 10^6.5^ recombinant PRVs with all codon deoptimization of US3-S and UL56 (Figure 8B). All mice inoculated with 10^6.5^ TCID_50_ with PRV^ΔTK&gE−AH02^ or recombinant PRVs presented slight inflammatory cell infiltration in the lung (Figure 8B). Moreover, it should be noted that no obvious histopathological changes were observed in the brains and lungs of mice inoculated with 10^5.5^ TCID_50_ and 10^4.5^ TCID_50_ with PRV^ΔTK&gE−AH02^ or recombinant PRVs. However, PRV^ΔTK&gE^-US3-S&UL56^T−CD^ displayed no further reduction in the pathogenicity to mice compared to PRV^ΔTK&gE^-US3-S^T−CD^ and PRV^ΔTK&gE^-UL56^T−CD^.

After 3 weeks of immunization, all the surviving mice were challenged with 100 LD_50_ PRV AH02LA strains. Notably, the protection efficiency was substantially provided by 10^6.5^ recombinant PRVs with codons deoptimizing US3-S or UL56, similar to 10^6.5^ PRV^ΔTK&gE−AH02^ (Table 1). Nonetheless, most mice inoculated with 10^5.5^ TCID_50_ and 10^4.5^ TCID_50_ PRV^ΔTK&gE−AH02^ or recombinant PRVs developed clinical signs of disease and died at 72–96 h post-challenge.

**Table 1:** Immunogenicity of the recoded viruses with codon deoptimization of US3-S or UL56 in mice.

**Virus strain**	**Doses**	**Numbers**	**Challenge**	
	**(TCID_50_)**		**Survival**	**Protection ratio**
PRV^ΔTK&gE^-US3-S^F10−CD^	10^6.5^	8	5	62.5%
	10^5.5^	8	0	0
	10^4.5^	8	0	0
PRV^ΔTK&gE^-UL56^F10−CD^	10^6.5^	8	4	50.0%
	10^5.5^	8	1	12.5%
	10^4.5^	8	0	0
PRV^ΔTK&gE^-US3-SandUL56^F10−CD^	10^6.5^	8	5	62.5%
	10^5.5^	8	1	12.5%
	10^4.5^	8	0	0
PRV^ΔTK&gE^-US3-S^T−CD^	10^6.5^	8	5	62.5%
	10^5.5^	8	0	0
	10^4.5^	8	0	0
PRV^ΔTK&gE^-UL56^T−CD^	10^6.5^	8	4	50.0%
	10^5.5^	8	0	0
	10^4.5^	8	0	0
PRV^ΔTK&gE^-US3-SandUL56^T−CD^	10^6.5^	8	6	75.0%
	10^5.5^	8	0	0
	10^4.5^	8	0	0
PRV^ΔTK&gE−AH02^	10^6.5^	8	5	62.5%
	10^5.5^	8	0	0
	10^4.5^	8	0	0
DMEM		8		

**Figure 9:**
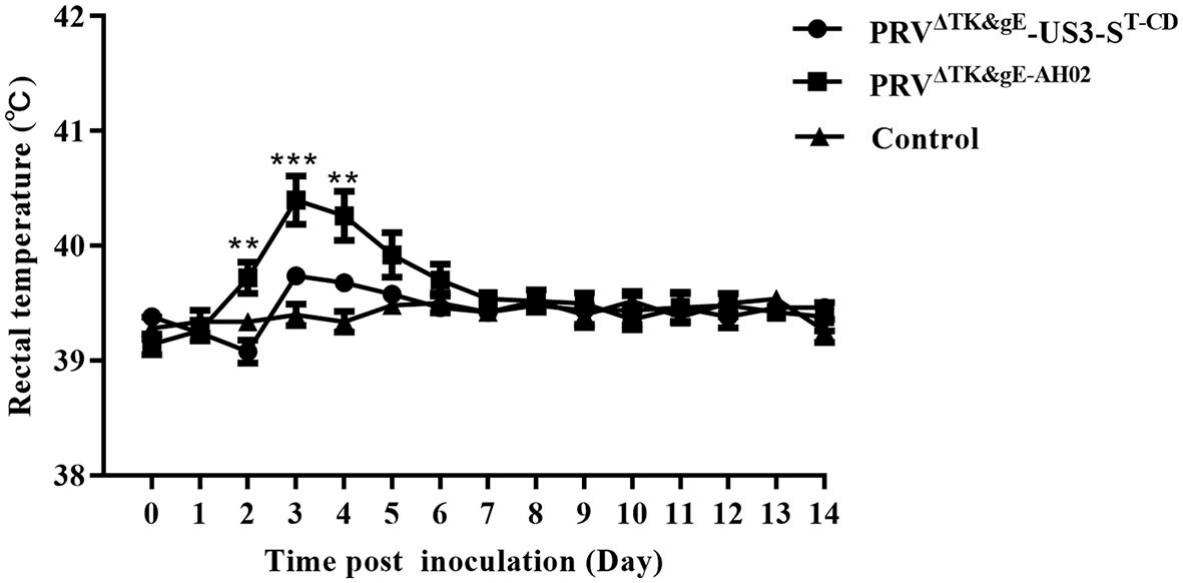
The graph shows the daily rectal temperatures of piglets post-inoculation. Data are presented as mean ± SEM. ** represents *P* < 0.01 and *** signifies *P* < 0.001.

**Figure 10:**
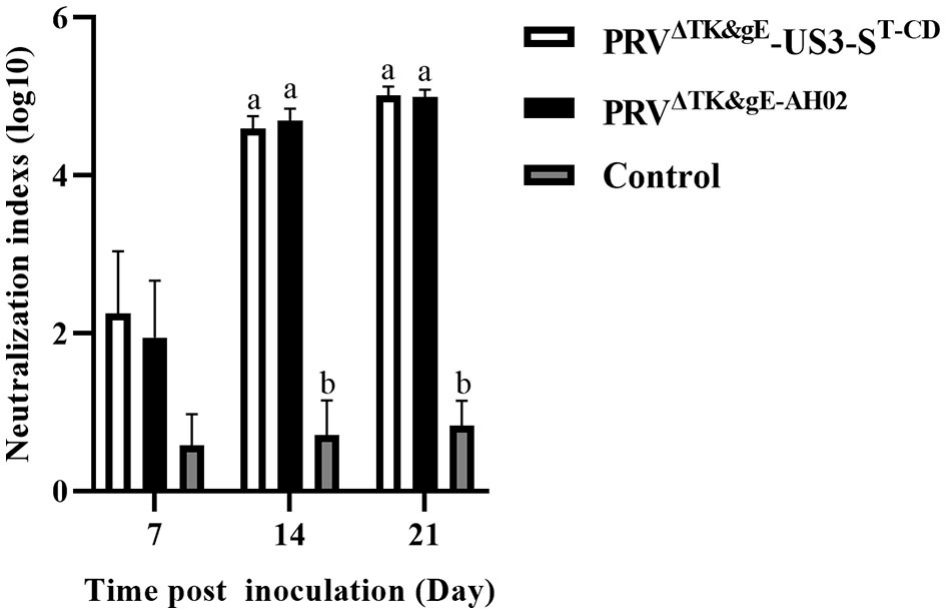
The graph presents the serum neutralization indices against PRV AH02LA strain in piglets at days 7, 14, and 21 post-inoculation. Letters (a, b) above the bars indicate statistical significance (*P* < 0.05) of serum neutralization indices among the three groups.

Considering the biosafety results in mice, we further performed the safety and immunogenicity checks in 1-day-old piglets. Among the treated piglets, two of five showed typical clinical symptoms of PRV infection from day 3 after the inoculation with PRV^ΔTK&gE−AH02^. In addition, the three piglets inoculated with PRV^ΔTK&gE−AH02^ showed higher body temperatures of 40.5°C on days 4 and 5 after inoculation (Figure 9). To this end, no clinical symptoms or body temperatures were observed in all piglets inoculated with PRV^ΔTK&gE^-US3-S^T−CD^. The ability of serum samples to neutralize PRV was detected after 7, 14, and 21 days of inoculation (Figure 10). Piglets vaccinated with PRV^ΔTK&gE^-US3-S^T−CD^ showed a high serum neutralization index. After 14 and 21 days of treatment, no significant difference was observed between piglets vaccinated with PRV^ΔTK&gE^-US3-S^T−CD^ and piglets vaccinated with PRV^ΔTK&gE−AH02^.

## 4. Discussion

Indeed, pseudorabies, caused by a variant PRV, has emerged as one of the most dreadful infections since 2011 in many Bartha-K61-vaccinated swine herds in China (An et al., 2013; Yu et al., 2014). Furthermore, it was increasingly recognized that the Bartha-K61 vaccine was incompletely protected by highly virulent PRV strains. Considering these challenges, attenuated PRV strains with gE/TK or gE/gI/TK deletions based on PRV variants have been constructed, showing excellent protection against the PRV challenge. Nonetheless, previous reports indicated that the safety of these gene deletion mutants was far from satisfactory (Wang et al., 2018; Xu et al., 2022). To develop a safe and effective live PRV vaccine, in this study, a codon deoptimization strategy was adapted to specifically target virulence genes US3 or UL56 based on the PRV gE/TK deletion strain. All codons deoptimizing US3-S or UL56 resulted in reduced target gene mRNA and protein expressions and decreased virus duplication. In addition, the stability of the recoded gene was confirmed by passaging in ST cells without new mutations. It was observed that all codons deoptimizing US3-S or UL56 based on the PRV gE/TK deletion strain caused attenuation of the recoded virus without affecting immunogenicity in mice and piglets.

Codon deoptimization offers several advantages for the generation of live-attenuated PRV vaccines. First, a live-attenuated vaccine with codon deoptimization contains many mutations that make viral reversion to parental virus extremely unlikely. In our study, we observed no reversion of the recombinant PRVs with codon deoptimization of US3-S or UL56 during serial passage on ST cells. However, serial passage in pigs was required to further evaluate the stability of codon-deoptimized PRV. Second, codon deoptimization shows no effect on the viral protein sequence, retaining the antigenicity of the virus identical to the parental virus. Third, the generation of live-attenuated PRV vaccines with codon deoptimization can be rapidly achieved by combining the synthesis of codon deoptimization genes with BAC technologies. Indeed, the PRV genome is double-stranded DNA that encodes for approximately 70 genes, containing both essential and non-essential genes. Since some non-essential genes often play important roles in PRV pathogenesis and host control, we speculate that their deoptimization may lead to reduced virulence without interfering with immunogenicity.

In the current study, *US3-S* and *UL56* genes were selected to explore the effect of codon deoptimization due to their involvement in viral virulence. Previous studies showed that the degree and position of codon deoptimization are inversely correlated with the degree of cytopathic effects and plaque size and are crucial to the degree of virus attenuation (Eschke et al., 2018; Lee et al., 2021). In this study, the first 10 codons (a small amount; first segment), or all codons (a large number; whole segment) of *US3-S* and *UL56* genes, were deoptimized to identify the effect of the degree and position of deoptimization on recoded expression and viral virulence. Nonetheless, the first 10 codon deoptimization of US3-S or UL56 showed no effect on the mRNA expression of the recoded gene, suggesting the degree and position of deoptimization could be chosen carefully in vaccine design. Furthermore, some essential genes are involved in viral egress, cell–cell spread, pathogenicity, and immunogenicity. Further studies involving codon deoptimizing essential genes should be attempted to develop a safe and effective PRV vaccine.

The molecular mechanisms of attenuation by codons deoptimizing US3-S and UL56 remain unknown. Previous studies indicated that codon deoptimization of genes in RNA viruses led to changes in RNA secondary structure, stability gene composition, and protein translation efficiency of the target gene (Kanaya et al., 2001; Knight et al., 2001; Burns et al., 2006; Meng et al., 2014). Moreover, the studies demonstrated that these were usually associated with decreased viral replication and virulence attenuation. In this study, all codon deoptimization negatively affected US3-S or UL56 RNA and protein levels after transient expression and RNA levels during virus replication. Therefore, protein translation efficiency might mediate the attenuation of recombinant PRVs with all codons deoptimizing US3-S and UL56.

Typically, successful live PRV vaccine candidates must show attenuation in the host while retaining immunogenicity. The recombinant PRVs with all codon deoptimization of US3-S or UL56 exhibited significantly reduced replication kinetics *in vitro* compared to the parental virus. Predictably, our *in vivo* data indicated that all codons deoptimizing US3-S or UL56 decreased virus load and attenuated pathological changes in the brains of mice. Surprisingly, PRV^ΔTK&gE^-US3-S&UL56^T−CD^ displayed no further reduction in pathogenicity to mice compared with PRV^ΔTK&gE^-US3-S^T−CD^ and PRV^ΔTK&gE^-UL56^T−CD^. Nonetheless, the potential mechanism remains unknown and needs further investigation. The protection efficiency provided by recombinant PRVs with codons deoptimizing US3-S or UL56 is similar to that of mice inoculated with their parental virus. Moreover, all codons deoptimizing US3-S caused attenuation of the recoded virus in piglets without loss of immunogenicity. However, future studies involving DNA loads and histological analysis in piglets are necessary to evaluate the pathogenicity of codon-deoptimized PRV in the major host. Furthermore, the safety and protective capacity of the other recoded virus in piglets also need further investigation to identify their potential use as a live vaccine candidate in pigs.

## 5. Conclusion

The codon deoptimization application in US3-S or UL56 based on PRV gE/TK deletion strain successfully generated six live recoded viruses. Among them, recombinant PRVs with all codon deoptimization of US3-S or UL56 cause virulence attenuation while retaining immunogenicity in mice and piglets. PRV^ΔTK&gE^-US3-S^T−CD^ showed good safety and a high serum neutralization index in piglets, which might be a promising vaccine candidate against PRV variants. Finally, our results indicated that codon deoptimization might be useful for attenuating PRV.

## Data availability statement

The datasets presented in this study can be found in online repositories. The names of the repository/repositories and accession number(s) can be found below: KM061380, OR228539, OR228540, KM061380, OR228541, and OR228542 (Genbank).

## Ethics statement

All animal experiment was approved by the Institutional Animal Care and Ethics Committee at the Jiangsu Academy of Agriculture Sciences [authorization number SYXK (Su) 2015-0019] and performed strictly with the guidelines provided by the Institutional Biosafety Committee. The studies were conducted in accordance with the local legislation and institutional requirements. Written informed consent was obtained from the owners for the participation of their animals in this study.

## Author contributions

CZ, RF, and JW conceived and designed the whole trial. CZ and MX wrote the manuscript. YL edited the manuscript. LZ constructed six live recoded viruses. MX, LZ, AG, SC, ZWe, YZ, LT, and ZWa conducted animal experiments and collected samples. All authors read and approved the final manuscript.

## References

[B1] An T. Q. Peng J. M. Tian Z. J. Zhao H. Y. Li N. Liu Y. M.. (2013). Pseudorabies virus variant in Bartha-K61-vaccinated pigs, China, 2012. Emerg. Infect. Dis. 19, 1749–1755. 10.3201/eid1911.130177 24188614 PMC3837674

[B2] Burns C. C. Shaw J. Campagnoli R. Jorba J. Vincent A. Quay J.. (2006). Modulation of poliovirus replicative fitness in HeLa cells by deoptimization of synonymous codon usage in the capsid region. J. Virol. 80, 3259–3272. 10.1128/JVI.80.7.3259-3272.2006 16537593 PMC1440415

[B3] Cai Y. Ye C. Cheng B. Nogales A. Iwasaki M. Yu S.. (2020). A Lassa fever live-attenuated vaccine based on codon deoptimization of the viral glycoprotein gene. mBio 11, e00039-20. 10.1128/mBio.00039-20 32098811 PMC7042690

[B4] Chomczynski P. Sacchi N. (1987). Single-step method of RNA isolation by acid guanidinium thiocyanate-phenol-chloroform extraction. Anal. Biochem. 162, 156–159. 10.1016/0003-2697(87)90021-2 2440339

[B5] Daniel G. R. Sollars P. J. Pickard G. E. Smith G. A. (2016). The pseudorabies virus protein, pUL56, enhances virus dissemination and virulence but is dispensable for axonal transport. Virology 488, 179–186. 10.1016/j.virol.2015.11.014 26655235 PMC4744496

[B6] Diaz-San Segundo F. Medina G. N. Ramirez-Medina E. Velazquez-Salinas L. Koster M. Grubman M. J.. (2016). Synonymous deoptimization of foot-and-mouth disease virus causes attenuation in vivo while inducing a strong neutralizing antibody response. J. Virol. 90, 1298–1310. 10.1128/JVI.02167-15 26581977 PMC4719607

[B7] Eschke K. Trimpert J. Osterrieder N. Kunec D. (2018). Attenuation of a very virulent Marek's disease herpesvirus (MDV) by codon pair bias deoptimization. PLoS Pathog. 14, e1006857. 10.1371/journal.ppat.1006857 29377958 PMC5805365

[B8] Freuling C. M. Muller T. F. Mettenleiter T. C. (2017). Vaccines against pseudorabies virus (PrV). Vet. Microbiol. 206, 3–9. 10.1016/j.vetmic.2016.11.019 27890448

[B9] Goncalves-Carneiro D. Bieniasz P. D. (2021). Mechanisms of attenuation by genetic recoding of viruses. mBio 12, e02238–e02220. 10.1128/mBio.02238-20 33402534 PMC8545087

[B10] Kanaya S. Yamada Y. Kinouchi M. Kudo Y. Ikemura T. (2001). Codon usage and tRNA genes in eukaryotes: correlation of codon usage diversity with translation efficiency and with CG-dinucleotide usage as assessed by multivariate analysis. J. Mol. Evol. 53, 290–298. 10.1007/s002390010219 11675589

[B11] Kimman T. G. De Wind N. De Bruin T. de Visser Y. Voermans J. (1994). Inactivation of glycoprotein gE and thymidine kinase or the US3-encoded protein kinase synergistically decreases in vivo replication of pseudorabies virus and the induction of protective immunity. Virology 205, 511–518. 10.1006/viro.1994.1672 7975253

[B12] Knight R. D. Freeland S. J. Landweber L. F. (2001). A simple model based on mutation and selection explains trends in codon and amino-acid usage and GC composition within and across genomes. Genome Biol. 2, RESEARCH0010. 10.1186/gb-2001-2-4-research0010 11305938 PMC31479

[B13] Lee M. H. P. Tan C. W. Tee H. K. Ong K. C. Sam I. C. Chan Y. F. (2021). Vaccine candidates generated by codon and codon pair deoptimization of enterovirus A71 protect against lethal challenge in mice. Vaccine. 39, 1708–1720. 10.1016/j.vaccine.2021.02.024 33640144

[B14] Lorenzo M. M. Nogales A. Chiem K. Blasco R. Martinez-Sobrido L. (2022). Vaccinia virus attenuation by codon deoptimization of the a24r gene for vaccine development. Microbiol. Spectr. 10, e0027222. 10.1128/spectrum.00272-22 35583360 PMC9241885

[B15] Lv J. Zhang C. Hou J. Fei R. Wang J. (2020a). Construction and biological characterization of pseudorabies virus variant of the inactivated US3 gene. Anim. Husb. Vet. Med. 52, 95–100.

[B16] Lv J. Zhang C. Wang Z. He Q. Wang J. Fei R. (2020b). Construction of UL43 and UL56 gene inactivation mutants of pseudorabies virus variant and analysis of their biological characteristics. Chin. J. Anim. Infect. Dis. 30, 34–41.

[B17] Meng J. Lee S. Hotard A. L. Moore M. L. (2014). Refining the balance of attenuation and immunogenicity of respiratory syncytial virus by targeted codon deoptimization of virulence genes. mBio 5, e01704–01714. 10.1128/mBio.01704-14 25249281 PMC4173764

[B18] Nogales A. Baker S. F. Ortiz-Riano E. Dewhurst S. Topham D. J. Martinez-Sobrido L. (2014). Influenza A virus attenuation by codon deoptimization of the NS gene for vaccine development. J. Virol. 88, 10525–10540. 10.1128/JVI.01565-14 24965472 PMC4178899

[B19] Olsen L. M. Ch'ng T. H. Card J. P. Enquist L. W. (2006). Role of pseudorabies virus Us3 protein kinase during neuronal infection. J. Virol. 80, 6387–6398. 10.1128/JVI.00352-06 16775327 PMC1488934

[B20] Pomeranz L. E. Reynolds A. E. Hengartner C. J. (2005). Molecular biology of pseudorabies virus: impact on neurovirology and veterinary medicine. Microbiol. Mol. Biol. Rev. 69, 462–500. 10.1128/MMBR.69.3.462-500.2005 16148307 PMC1197806

[B21] Sehl J. Portner S. Klupp B. G. Granzow H. Franzke K. Teifke J. P.. (2020). Roles of the different isoforms of the pseudorabies virus protein kinase pUS3 in nuclear egress. J. Virol. 94, e02029–e02019. 10.1128/JVI.02029-19 31941788 PMC7081916

[B22] Tischer B. K. Smith G. A. Osterrieder N. (2010). En passant mutagenesis: a two step markerless red recombination system. Methods Mol. Biol. 634, 421–430. 10.1007/978-1-60761-652-8_30 20677001

[B23] Wang J. Song Z. Ge A. Guo R. Qiao Y. Xu M.. (2018). Safety and immunogenicity of an attenuated Chinese pseudorabies variant by dual deletion of TKandgE genes. BMC. Vet. Res. 14, 287. 10.1186/s12917-018-1536-7 30241529 PMC6150974

[B24] Weng M. Guo Z. Lu Q. Jin Q. Jiang Y. Wang F.. (2023). Pseudorabies virus regulates the extracellular translocation of annexin A2 to promote its proliferation. *J*. Virol. 97, e0154522. 10.1128/jvi.01545-22 36786600 PMC10062141

[B25] Xu M. Zhang C. Liu Y. Chen S. Zheng Y. Wang Z.. (2022). A noval strategy of deletion in PK gene for construction of a vaccine candidate with exellent safety and complete protection efficiency against high virulent Chinese pseudorabies virus variant. Virus Res. 313, 198740. 10.1016/j.virusres.2022.198740 35271886

[B26] Yu X. Zhou Z. Hu D. Zhang Q. Han T. Li X.. (2014). Pathogenic pseudorabies virus, China, 2012. Emerg. Infect. Dis. 20, 102–104. 10.3201/eid2001.130531 24377462 PMC3884716

[B27] Zhang C. Guo L. Jia X. Wang T. Wang J. Sun Z.. (2015). Construction of a triple gene-deleted Chinese Pseudorabies virus variant and its efficacy study as a vaccine candidate on suckling piglets. Vaccine 33, 2432–2437. 10.1016/j.vaccine.2015.03.094 25865469

[B28] Zhang C. Liu Y. Chen S. Qiao Y. Guo M. Zheng Y.. (2019a). A gDandgC-substituted pseudorabies virus vaccine strain provides complete clinical protection and is helpful to prevent virus shedding against challenge by a Chinese pseudorabies variant. BMC. Vet. Res. 15, 2. 10.1186/s12917-018-1766-8 30606159 PMC6318912

[B29] Zhang C. Liu Y. Chen S. Qiao Y. Zheng Y. Xu M.. (2019b). Effects of intranasal pseudorabies virus AH02LA infection on microbial community and immune status in the ileum and colon of piglets. Viruses 11, 518. 10.3390/v11060518 31195631 PMC6631256

[B30] Zhou J. Li S. Wang X. Zou M. Gao S. (2017). Bartha-k61 vaccine protects growing pigs against challenge with an emerging variant pseudorabies virus. Vaccine 35, 1161–1166. 10.1016/j.vaccine.2017.01.003 28131396

