# Supplementary material for: Construction of pseudorabies virus variant attenuated vaccine: codon deoptimization of US3 and UL56 genes based on PRV gE/TK deletion strain
Source: Front Microbiol. 2023 Oct 10;14:1248573. doi: 10.3389/fmicb.2023.1248573 (PMC10595036; doi:10.3389/fmicb.2023.1248573)
Supplement: Supplementary file 1 [file Data_Sheet_1.pdf]

Supplementary Material

Construction of Pseudorabies Virus (PRV) Variant Attenuated Vaccine: Codon Deoptimization of US3 and UL56 Gene Based on PRV gE/TK Deletion Strain

Mengwei Xu[1,2,3,4†], Laixu Zhu[1,2,3,4†], Aimin Ge[5], Yamei Liu[1,2,3], Saisai Chen[1,2,3], Ziwen Wei[1,2,3], Yating Zheng[1,2,3], Ling Tong[1,2,3], Zhisheng Wang[1,2,3], Rongmei Fei[4], Jichun Wang[1,2,3*], Chuanjian Zhang[1,2,3*]

[1]National Research Center of Engineering and Technology for Veterinary Biologicals, Jiangsu Key Laboratory for Food Quality and Safety-State Key Laboratory Cultivation Base of the Ministry of Science and Technology, Institute of Veterinary Immunology and Engineering, Jiangsu Academy of Agricultural Sciences, Nanjing 210014, China

[2]GuoTai (Taizhou) Center of Technology Innovation for Veterinary Biologicals, Taizhou 225300, China

[3]Jiangsu Co-Innovation Center for Prevention and Control of Important Animal Infectious Diseases and Zoonoses, Yangzhou 225009, China

[4]College of Veterinary Medicine, Nanjing Agricultural University, Nanjing 210095, China; 2020107043@stu.njau.edu.cn

[5]Shandong Vocational Animal Science and Veterinary College, Weifang 261061, China

*** Correspondence:** Jichun Wang; jcwang@263.net; Chujian Zhang; E-mail: zcj6717855@126.com

Supplementary Information

Supplementary Information S1 Nucleotide sequence of US3-S (accession numbers: KM061380), US3-S$^{F10\text{-}CD}$ (accession numbers: OR228539), US3-S$^{T\text{-}CD}$ (accession numbers: OR228540), UL56 (accession numbers: KM061380), UL56$^{F10\text{-}CD}$ (accession numbers: OR228541) and UL56$^{T\text{-}CD}$ (accession numbers: OR228542). Nucleotide changes compared to wide type are highlighted in red.

US3-S

ATGGCCGACGCCGGAATCCCCGACGAGATCCTGTACTCGGACATCAGCGACGACGAGAT
CATCATCGACGGCGACGGCGACAGCAGCGGGGACGAGGACACCGACGATGACGGGGGG
CTGACGCGGCAGGCCGCGTCGCGCATCGCCACGGACCTGGGCTTCGAGGTGCTGCAGCC
CCTGCAGTCGGGCTCGGAGGGCCGCGTCTTCGTGGCCCGCCGGCCCGGCGAGGCGGACA
CGGTGGTGCTGAAGGTGGGCCAGAAGCCCTCGACGCTGATGGAGGGCATGCTGCTGAA
GCGCCTGGCCCACGATAACGTCATGAGCCTGAAGCAGATGCTCGCCCGGGGCCCGGTGA
CGTGCCTGGTCCTGCCGCACTTTCGGTGCGATCTGTACAGCTACCTGACCATGCGGGACG
GGCCGCTGGACATGCGCGACGCCGGCCGCGTGATCCGTTCCGTGCTCCGCGGGCTCGCC
TACCTGCACGGGATGCGCATCATGCACCGCGACGTCAAGGCGGAGAACATCTTCCTCGA
GGACGTGGACACGGTGTGCCTGGGGGACCTCGGGGCCGCGCGCTGCAACGTGGCGGCG
CCCAACTTTTACGGGCTCGCCGGGACCATCGAGACCAACGCCCCCGAGGTGCTCGCGCG
CGACCGCTACGACACCAAGGTCGACGTCTGGGGTGCGGGGGTGGTGCTCTTCGAGACGC
TGGCCTACCCCAAGACGATCACCGGCGGGGACGAGCCCGCGATCAACGGGGAGATGCA
CCTGATCGACCTCATCCGCGCCCTCCGCGGGGTGCACCCCGAGGAGTTCCCGCCCGACA
CGCGCCTCCGGAGCGAGTTCGTCCGGTACGCCGGGACCCATCGCCAGCCGTACACGCAG
TACGCGCGCGTGGCTCGCCTCGGGCTGCCCGAGACGGGGGCTTTCCTGATTTACAAGAT
GTTGACGTTTGATCCCGTCCGCCGCCCTTCCGCTGATGAGATACTCAACTTTGGAATGTG
GACCGTATAA

US3-S$^{F10\text{-}CD}$

ATGGCGGATGCGGGTATACCGGATGAAATACTGTACTCGGACATCAGCGACGACGAGAT
CATCATCGACGGCGACGGCGACAGCAGCGGGGACGAGGACACCGACGATGACGGGGGG
CTGACGCGGCAGGCCGCGTCGCGCATCGCCACGGACCTGGGCTTCGAGGTGCTGCAGCC
CCTGCAGTCGGGCTCGGAGGGCCGCGTCTTCGTGGCCCGCCGGCCCGGCGAGGCGGACA
CGGTGGTGCTGAAGGTGGGCCAGAAGCCCTCGACGCTGATGGAGGGCATGCTGCTGAA
GCGCCTGGCCCACGATAACGTCATGAGCCTGAAGCAGATGCTCGCCCGGGGCCCGGTGA

CGTGCCTGGTCCTGCCGCACTTTCGGTGCGATCTGTACAGCTACCTGACCATGCGGGACG
GGCCGCTGGACATGCGCGACGCCGGCCGCGTGATCCGTTCCGTGCTCCGCGGGCTCGCC
TACCTGCACGGGATGCGCATCATGCACCGCGACGTCAAGGCGGAGAACATCTTCCTCGA
GGACGTGGACACGGTGTGCCTGGGGGACCTCGGGGCCGCGCGCTGCAACGTGGCGGCG
CCCAACTTTTACGGGCTCGCCGGGACCATCGAGACCAACGCCCCCGAGGTGCTCGCGCG
CGACCGCTACGACACCAAGGTCGACGTCTGGGGTGCGGGGGTGGTGCTCTTCGAGACGC
TGGCCTACCCCAAGACGATCACCGGCGGGGACGAGCCCGCGATCAACGGGGAGATGCA
CCTGATCGACCTCATCCGCGCCCTCCGCGGGGTGCACCCCGAGGAGTTCCCGCCCGACA
CGCGCCTCCGGAGCGAGTTCGTCCGGTACGCCGGGACCCATCGCCAGCCGTACACGCAG
TACGCGCGCGTGGCTCGCCTCGGGCTGCCCGAGACGGGGGCTTTCCTGATTTACAAGAT
GTTGACGTTTGATCCCGTCCGCCGCCCTTCCGCTGATGAGATACTCAACTTTGGAATGTG
GACCGTATAA

US3-S$^{T\text{-}CD}$

ATGGCGGATGCGGGTATACCGGATGAAATACTATATTCGGATATAAGTGATGATGAAAT
AATAATAGATGGTGATGGTGATAGTAGTGGTGATGAAGATACGGATGATGATGGTGGTC
TAACGCGTCAAGCGGCGTCGCGTATAGCGACGGATCTAGGTTTTGAAGTACTACAACCC
GTGCAATCGGGTTCGGAAGGTCGTGTATTTGTAGCGCGTCGTCCGGGTGAAGCGGATAC
GGTAGTACTAAAAGTAGGTCAAAAACCGTCGTCGCTAATGGAAGGTATGCTACTAAAAC
GTCTAGCGCATGTAAATGTAATGAGTCTAAAACAAATGCTAGCGCGTGGTCCGGTAACG
TGTCTAGTACTACCGCATTTTCGTTGTGATCTATATAGTTATCTAACGATGCGTGATGGT
CCGCTAGATATGCGTGATGCGGGTCGTGTAATACGTTCGGTACTACGTGGTCTAGCGTAT
CTACATGGTATGCGTATAATGCATCGTGATGTAAAAGCGGAAAATATATTTCTAGAAGA
TGTAGATACGGTATGTCTAGGTGATCTAGGTGCGGCGCGTTGTAATGTAGCGGCGCCGA
ATTTTTATGGTCTAGCGGGTACGATAGAAACGAATGCGCCGGAAGTACTAGCGCGTGAT
CGTTATGATACGAAAGTAGATGTATGGGGTGCGGGTGTAGTACTATTTGAAACGCTAGC
GTATCCGAAAACGATAACGGGTGGTGATGAACCGGCGATAAATGGTGAAATGCATCTA
ATAGATCTAATACGTGCGCTACGTGGTGTACATCCGGAAGAATTTCCGCCGGATACGCG
TCTACGTAGTGAATTTGTACGTTATGCGGGTACGCATCGTCAACCGTATACGCAATATGC
GCGTGTAGCGCGTCTAGGTCTACCGGAAACGGGTGCGTTTCTAATATATAAAATGTTAA
CGTTTGATCCGGTACGTCGTCCGTCGGCGGATGAAATACTAAATTTTGGTATGTGGACGG
TATAA

UL56

ATGCCTCCACAACGAGCCCGCGGGGCTCCGCCGCGCCGTCGCGGCAGCGACCCGCCCGA
TCCAGGCAGCCTCGCCGGGCGGCCCTCGCCCGGGGGGAGAGGAGGCGGAGGGCAGCGC
CGCCCCCTCTCGCGCAGCAGCTCGCTCACGTCCGTCGCCTCGGCGCCCGTGGAGACGCC
CGTCGTCGCGGAGGCTCCGGGGCTCGGCGCCCCCGGCTCCAGGCCGCCCTCCTACGGGG
ACGTCGTCCGCGTCGGGCCGCGCCCGCACCGATCGCCGGACACGCCGCTGTTTGCCCGG
GGCCCGCCCCCGTCCTACTCGGAGACGCTCCTGTTCGACCCGCCCGCGTACGCGGTGAC

CATCCCGGACCCGCCGGCGTACGAGCCCACCGTCATCGGGCCGCACCCGCCGCGCCCCC
GCGACTGGATCTCCTCGCCCTCGGTGGTGCAGCCGTCGCTGCTGGGCCCCTTCAGCCAGT
GCCTCCCGCGGGTGACCTGCCCCGACTGCCGCTACCCCGAAGACCGCCCGATGGTGCTC
GTGGGCTTCCTCTGGGGGGGACTGCTCCTGCTGGTGGGCCTCGTGTTTCTGATCCTGCTC
CCGGTGCTCCGGGAGTCCGTCGTGTTTCCCTGA

UL56$^{\text{F10-CD}}$

ATGCCGCCGCAACGTGCTCGTGGTGCGCCGCCGCGCCGTCGCGGCAGCGACCCGCCCGA
TCCAGGCAGCCTCGCCGGGCGGCCCTCGCCCGGGGGGAGAGGAGGCGGAGGGCAGCGC
CGCCCCCTCTCGCGCAGCAGCTCGCTCACGTCCGTCGCCTCGGCGCCCGTGGAGACGCC
CGTCGTCGCGGAGGCTCCGGGGCTCGGCGCCCCCGGCTCCAGGCCGCCCTCCTACGGGG
ACGTCGTCCGCGTCGGGCCGCGCCCGCACCGATCGCCGGACACGCCGCTGTTTGCCCGG
GGCCCGCCCCCGTCCTACTCGGAGACGCTCCTGTTCGACCCGCCCGCGTACGCGGTGAC
CATCCCGGACCCGCCGGCGTACGAGCCCACCGTCATCGGGCCGCACCCGCCGCGCCCCC
GCGACTGGATCTCCTCGCCCTCGGTGGTGCAGCCGTCGCTGCTGGGCCCCTTCAGCCAGT
GCCTCCCGCGGGTGACCTGCCCCGACTGCCGCTACCCCGAAGACCGCCCGATGGTGCTC
GTGGGCTTCCTCTGGGGGGGACTGCTCCTGCTGGTGGGCCTCGTGTTTCTGATCCTGCTC
CCGGTGCTCCGGGAGTCCGTCGTGTTTCCCTGA

UL56$^{\text{T-CD}}$

ATGCCGCCGCAACGTGCTCGTGGTGCGCCGCCGCGTCGTCGTGGTAGTGATCCGCCGGA
TCCGGGTAGTCTAGCGGGTCGTCCGTCGCCGGGTGGTAGAGGTGGTGGTGGTCAACGTC
GTCCGCTATCGCGTAGTAGTTCGCTAACGTCGGTAGCGTCGGCGCCGGTAGAAACGCCG
GTAGTAGCGGAAGCGCCGGGTCTAGGTGCGCCGGGTTCGAGACCGCCGTCGTATGGTGA
TGTAGTACGTGTAGGTCCGCGTCCGCATCGTTCGCCGGATACGCCGCTATTTGCGCGTGG
TCCGCCGCCGTCGTATTCGGAAACGCTACTATTTGATCCGCCGGCGTATGCGGTAACGAT
ACCGGATCCGCCGGCGTATGAACCGACGGTAATAGGTCCGCATCCGCCGCGTCCGCGTG
ATTGGATATCGTCGCCGTCGGTAGTACAACCGTCGCTACTAGGTCCGTTTAGTCAATGTC
TACCGCGTGTAACGTGTCCGGATTGTCGTTATCCGGAAGATCGTCCGATGGTACTAGTAG
GTTTTCTATGGGGTGGTCTACTACTACTAGTAGGTCTAGTATTTCTAATACTACTACCGG
TACTACGTGAATCGGTAGTATTTCCGTGA

Supplementary Tables

Supplementary Table S1 Primers for PCR, sequencing or RT-PCR

Primer	Sequence (5'- 3')
US3-S ins mKate2 F	AGATCTCGAGCTCAAGCTTCGAATTCATGGCCGACGCC GGAATCCC
US3-S ins mKate2 R	CCGCGGTACCGTCGACTGCAGAATTCTACGGTCCACAT TCCAA
US3-S^{F10-CD} ins mKate2 F	AGATCTCGAGCTCAAGCTTCGAATTCATGGCGGATGCG GGTATACC
US3-S^{F10-CD} ins mKate2 R	CCGCGGTACCGTCGACTGCAGAATTCTACGGTCCACAT TCCAA
US3-S^{T-CD} ins mKate2 F	AGATCTCGAGCTCAAGCTTCGAATTCATGGCGGATGCG GGTATACC
US3-S^{T-CD} ins mKate2 R	CCGCGGTACCGTCGACTGCAGAATTCTACCGTCCACAT ACCAA
UL56 ins mKate2 F	AGATCTCGAGCTCAAGCTTCGAATTCATGCCTCCACAA CGAGCCCG
UL56 ins mKate2 R	CCGCGGTACCGTCGACTGCAGAATTCGGGAAACACGAC GGACT
UL56^{F10-CD} ins mKate2 F	AGATCTCGAGCTCAAGCTTCGAATTCATGCCGCCGCAA CGTGCTCG
UL56^{F10-CD} ins mKate2 R	CCGCGGTACCGTCGACTGCAGAATTCGGGAAACACGAC GGACT
UL56^{T-CD} ins mKate2 F	AGATCTCGAGCTCAAGCTTCGAATTCATGCCGCCGCAA CGTGCTCG
UL56^{T-CD} ins mKate2 R	CCGCGGTACCGTCGACTGCAGAATTCCGGAAATACTAC CGATT
KAN ins US3-S^{F10-CD} F	CCCCCGGGGCCCGGTGACGTGCCTGGTCCTGCCGCACT TTCGGTGCGAGGATGACGACGATAAGTAGGGATAAC
KAN ins US3-S^{F10-CD} R	CCCCCGGGGGGTAATGCCAGTGTTACAACCA
KAN ins US3-S^{T-CD} F	TGCTCTAGAAGATGTAGATACGGTATGTCTAGGTGATC TAGGTGCGGCGGGATGACGACGATAAGTAGGGATAAC
KAN ins US3-S^{T-CD} R	TGCTCTAGAGGGTAATGCCAGTGTTACAACCA
KAN ins UL56^{F10-CD} F	GGGCCGGCGTACGAGCCCACCGTCATCGGGCCGCACCC GCCGCGCCCCGGATGACGACGATAAGTAGGGATAAC
KAN ins UL56^{F10-CD} R	GGGCCGGCGGGTAATGCCAGTGTTACAACCA
KAN ins UL56^{T-CD} F	CCGGATATCGTCGCCGTCGGTAGTACAACCGTCGCTAC TAGGTCCGTTTGGATGACGACGATAAGTAGGGATAAC
KAN ins UL56^{T-CD} R	CCGGATATCGGGTAATGCCAGTGTTACAACCA
US3-S^{F10-CD} En pa F	GTTGTCGCGCGTCCACGCCCAGCGCTCGCACGCAGCAA CAATGGCGGATGCGGGTATACC

US3-S^{F10-CD} En pa R	GCAAAGGTGTGTGTGTCCTACCGCTCGGAGCCGGGCCG TTTTATACGGTCCACATTCCAA
US3-S^{T-CD} En pa F	GTTGTCGCGCGTCCACGCCCAGCGCTCGCACGCAGCAA CAATGGCGGATGCGGGTATACC
US3-S^{T-CD} En pa R	GCAAAGGTGTGTGTGTCCTACCGCTCGGAGCCGGGCCG TTTTATACCGTCCACATACCAA
UL56^{F10-CD} En pa F	GGGCACCGCCGTCCGCCGCCAGCCGCCGTGGGAGGCAG ACATGCCGCCGCAACGTGCTCG
UL56^{F10-CD} En pa R	GACGCGAATATCGATGGGGCGGGCGAGCCGAGTTTATT GATCAGGGAAACACGACGGACT
UL56^{T-CD} En pa F	GGGCACCGCCGTCCGCCGCCAGCCGCCGTGGGAGGCAG ACATGCCGCCGCAACGTGCTCG
UL56^{T-CD} En pa R	GACGCGAATATCGATGGGGCGGGCGAGCCGAGTTTATT GATCACGGAAATACTACCGATT
US3-S check F	GGAGATGGGTCACCAAGAGG
US3-S check R	AGATGCGCAAAGGTGTGTGT
UL56 check F	CCGCCACCCTGGGTATTAAC
UL56 check R	GCCGAGTTTATTGATCAGGG
H1-H2-gI-ΔgE F	GTACCCGTACACCGAGTCGT
H1-H2-gI-ΔgE R	TTGTGGACCCGCGCGAACAT
PRV ΔgE check F	AGCCCCGGGAAGATAGCCAT
PRV ΔgE check R	ATCGCGGAACCAGACGTCGAAG
US3-S mRNA F	GTCGGGCTCGGAGGGCCGCG
US3-S mRNA R	CGGGCCCCGGGCGAGCATCT
US3-S^{T-CD} mRNA F	ATCGGGTTCGGAAGGTCGTG
US3-S^{T-CD} mRNA R	CGGACCACGCGCTAGCATTT
UL24 mRNA F	CTTCCCCCGGAACCTCAACA
UL24 mRNA R	TGGGCCACAAACACCAGCAG
UL40 mRNA F	AGCGCCATCCAGCTGATGCT
UL40 mRNA R	GCGAACGAGGAGGCGAAGAA
UL44 mRNA F	CGCCTTCGTGACCAACAGCA
UL44 mRNA R	GTACCACACGGCCTCGCAGC
UL52 mRNA F	TTGAAGTGCGCGTCGAGGTG
UL52 mRNA R	AGCTTCTTCGAGCGCAAGGC
UL56 mRNA F	GCAGCGCCGCCCCCTCTCGC
UL56 mRNA R	CGGGCGCGGCCCGACGCGGA
UL56^{T-CD} mRNA F	TCAACGTCGTCCGCTATCGC
UL56^{T-CD} mRNA R	CGGACGCGGACCTACACGTA
gB mRNA F	GTCCGTGAAGCGGTTCGTGAT
gB mRNA R	ACAAGTTCAAGGCCCACATCTAC

Supplementary Table S2 The titer of recombinant viruses with US3-S and UL56 codon deoptimization at each time point post-infection

Hours post infection	Virus titer[1] (lgTCID$_{50}$/mL)							
	PRV$^{\Delta TK\&gE}$-US3-S$^{F10\text{-}CD}$	PRV$^{\Delta TK\&gE}$-UL56$^{F10\text{-}CD}$	PRV$^{\Delta TK\&gE}$-US3-S&UL56$^{F10\text{-}CD}$	PRV$^{\Delta TK\&gE}$-US3-S$^{T\text{-}CD}$	PRV$^{\Delta TK\&gE}$-UL56$^{T\text{-}CD}$	PRV$^{\Delta TK\&gE}$-US3-S&UL56$^{T\text{-}CD}$	PRV$^{\Delta TK\&gE\text{-}AH02}$	*P*-value
6	1.65 ± 0.05^{a}	0.00 ± 0.00^{b}	0.00 ± 0.00^{b}	0.00 ± 0.00^{b}	1.07 ± 0.53^{ab}	0.53 ± 0.53^{b}	0.00 ± 0.00^{b}	0.005
12	2.55 ± 0.10^{b}	1.65 ± 0.05^{c}	1.78 ± 0.12^{c}	0.00 ± 0.00^{d}	2.33 ± 0.08^{b}	2.38 ± 0.07^{b}	3.13 ± 0.20^{a}	< 0.001
24	4.38 ± 0.62^{b}	5.20 ± 0.33^{ab}	5.20 ± 0.60^{ab}	4.13 ± 0.31^{b}	4.20 ± 0.25^{b}	4.62 ± 0.22^{b}	6.17 ± 0.08^{a}	0.026
36	6.32 ± 0.04^{bc}	7.08 ± 0.30^{a}	6.78 ± 0.12 ^{ab}	5.70 ± 0.05^{d}	6.17 ± 0.10^{bc}	6.55 ± 0.10^{bc}	6.81 ± 0.12^{ab}	0.006
48	6.69 ± 0.08^{b}	7.41 ± 0.05^{a}	6.77 ± 0.27^{b}	7.00 ± 0.14^{ab}	6.86 ± 0.19^{b}	7.00 ± 0.14^{ab}	7.43 ± 0.03^{a}	0.019
60	7.00 ± 0.14^{b}	6.86 ± 0.22^{b}	7.58 ± 0.08^{a}	6.35 ± 0.08^{c}	6.08 ± 0.22^{c}	6.58 ± 0.04^{bc}	7.08 ± 0.17^{b}	< 0.001
72	6.05 ± 0.13^{bc}	6.45 ± 0.23^{b}	6.92 ± 0.17^{a}	5.92 ± 0.08^{c}	5.70 ± 0.15^{cd}	6.38 ± 0.22^{b}	6.99 ± 0.20^{a}	< 0.001

[1] ST cells are infected with PRV$^{\Delta TK\&gE\text{-}AH02}$ and its six mutants at an MOI of 0.01. At 6, 12, 24, 36, 48, 60, and 72 h post-infection, the culture cells are harvested and titrated in ST cells.

$^{a, b, c, d}$ indicates statistical significance ($P < 0.05$) of virus titer among the 7 viruses.

Supplementary Figure

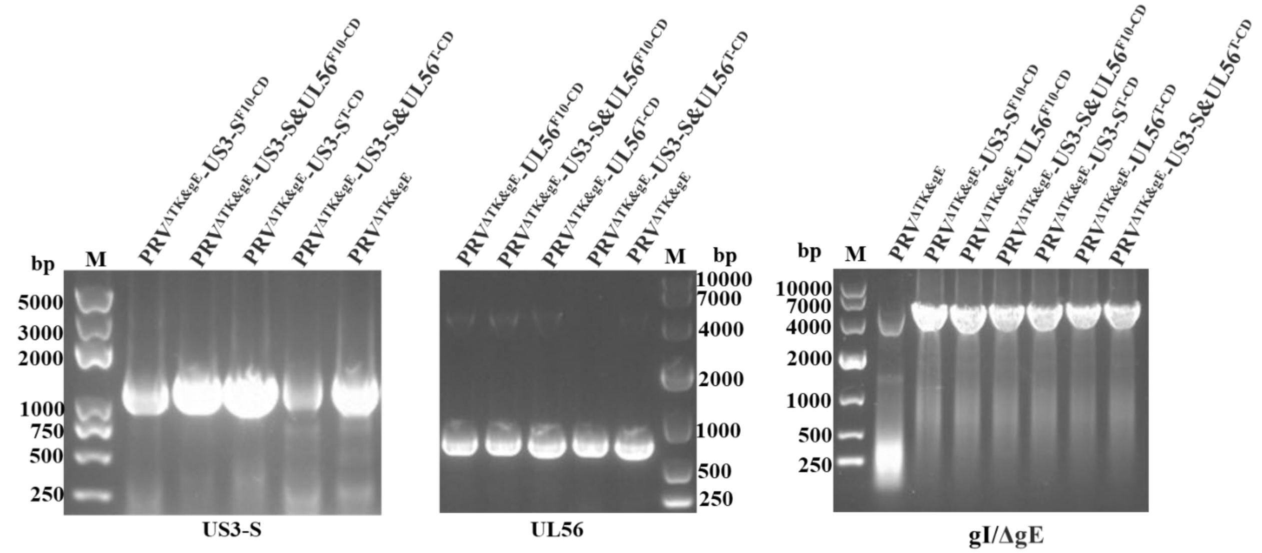

Supplementary Figure S1 Stability of recombinant PRVs with gI-ΔgE and the recoded US3-S and UL56 genes. The recombinant viruses were passaged 20 times on ST cells, gI-ΔgE and recoded US3-S and UL56 were detected by PCR.
